# DENTINE SIALOPHOSPHOPROTEIN SIGNAL IN DENTINEOGENESIS AND DENTINE REGENERATION

**DOI:** 10.22203/eCM.v042a04

**Published:** 2021-07-18

**Authors:** M.M. Liu, W.T. Li, X.M. Xia, F. Wang, M. MacDougall, S. Chen

**Affiliations:** 1 Department of Developmental Dentistry, School of Dentistry, the University of Texas Health Science Center at San Antonio, San Antonio, TX 78229, USA; 2 Department of Endodontics, School of Stomatology, Tongji University, Shanghai, 200072, China; 3 Department of Pathology, Weifang Medical University, Weifang, 261053, China; 4 Department of Obstetrics and Gynaecology, Second Xiangya Hospital, Central South University Changsha, 410011, China; 5 Department of Anatomy, Fujian Medical University, Fuzhou, 350122, China; 6 UBC Faculty of Dentistry, University of British Columbia, Vancouver, BC, V6T 1Z3, Canada

**Keywords:** Dentine, dental mesenchymal stem cells, dental caries, dentine regeneration, small integrin-binding ligand N linked glycoproteins, dentine sialoprotein, dentine glycoprotein, dentine phosphoprotein, dentine, dentine sialophosphoprotein

## Abstract

Dentineogenesis starts on odontoblasts, which synthesise and secrete non-collagenous proteins (NCPs) and collagen. When dentine is injured, dental pulp progenitors/mesenchymal stem cells (MSCs) can migrate to the injured area, differentiate into odontoblasts and facilitate formation of reactionary dentine. Dental pulp progenitor cell/MSC differentiation is controlled at given niches. Among dental NCPs, dentine sialophosphoprotein (DSPP) is a member of the small integrin-binding ligand N-linked glycoprotein (SIBLING) family, whose members share common biochemical characteristics such as an Arg-Gly-Asp (RGD) motif. DSPP expression is cell- and tissue-specific and highly seen in odontoblasts and dentine. DSPP mutations cause hereditary dentine diseases. DSPP is catalysed into dentine glycoprotein (DGP)/sialoprotein (DSP) and phosphoprotein (DPP) by proteolysis. DSP is further processed towards active molecules.

DPP contains an RGD motif and abundant Ser-Asp/Asp-Ser repeat regions. DPP-RGD motif binds to integrin αVβ3 and activates intracellular signalling *via* mitogen-activated protein kinase (MAPK) and focal adhesion kinase (FAK)-ERK pathways. Unlike other SIBLING proteins, DPP lacks the RGD motif in some species. However, DPP Ser-Asp/Asp-Ser repeat regions bind to calcium-phosphate deposits and promote hydroxyapatite crystal growth and mineralisation *via* calmodulin-dependent protein kinase II (CaMKII) cascades.

DSP lacks the RGD site but contains signal peptides. The tripeptides of the signal domains interact with cargo receptors within the endoplasmic reticulum that facilitate transport of DSPP from the endoplasmic reticulum to the extracellular matrix. Furthermore, the middle- and COOH-terminal regions of DSP bind to cellular membrane receptors, integrin β6 and occludin, inducing cell differentiation. The present review may shed light on DSPP roles during odontogenesis.

## Introduction

**Fig. 1.**
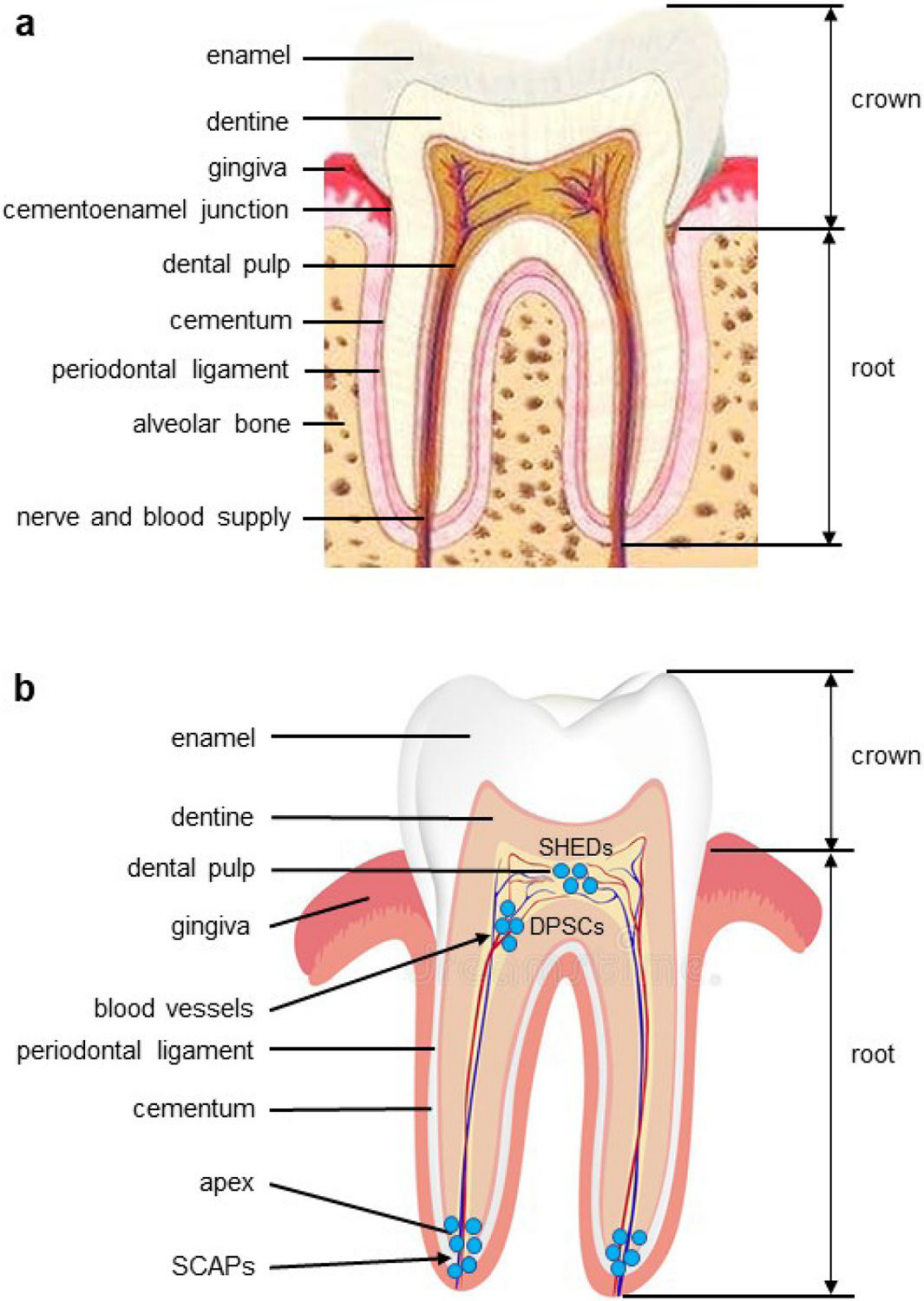
Schematic representation of a molar and MSCs found in the teeth. (**a**) The crown of the tooth is covered with enamel, while the root is covered with cementum. The cementoenamel junction is located at the enamel and root. The root is surrounded by the alveolar bone through periodontal ligaments. The dentine surrounds the dental pulp. Nerves and blood vessels enter the dental pulp from the apical foramen of the tooth and provide nutrition and innervation to odontoblasts and dental pulp. (**b**) DPSCs; SCAPs; SHEDs

The tooth is a highly mineralised organ resulting from the interactions between the dental oral epithelial and mesenchymal cells. It is composed of enamel, dentine, cementum, soft connective tissues and periodontium (Fig. 1a) (Mitsiadis et al., 2015; Nanci, 2012). The dentine is a thick highly mineralised tissue layer (present underneath the enamel) consisting of dentinal tubules and inter-tubular dentine and acts as a secondary barrier against infections of the dental pulp cavity (Lopez-Cazaux et al., 2006). Dentineogenesis starts at the onset of odontoblast differentiation. Odontoblasts originate from neural-crest-derived mesenchymal cells, which differentiate to form odontoblasts in specific temporal-spatial patterns, originating at the principal cusp tip and advancing toward the base of the teeth (Chen et al., 2008; Thesleff, 2003). Odontoblasts are mitotic cells organised as a layer of barrier cells along the edge between the dentine and dental pulp cavity. Odontoblasts synthesise and secrete the organic ECM proteins (Linde and Goldberg, 1993; MacDougall et al., 1997). Dentine is composed mostly of HA (70 % by weight), ~ 12 % water as well as collagens and NCPs (Linde and Goldberg, 1993; MacDougall et al., 1997). Odontoblasts in odontogenesis and dental caries participate in the physiological primary and secondary dentine formation. Also, odontoblasts maintain the dentine metabolism throughout the life of the tooth and serve as the first line of defence against dentine pathogen invasion by RD (reparative, tertiary) formation at the dentine-pulp interface beneath the carious infected dentine region (Couve et al., 2014).

The dental pulp is a loose connective tissue and contains blood vessels with abundant capillaries and an innervated tissue under the odontoblast layer. Blood vessels facilitate the exchange of nutrients and waste products in the dental pulp (Lopez-Cazaux et al., 2006; Tziafas et al., 2000). DPCs are a heterogeneous population retaining a source of MSCs (Tirino et al., 2012). Maintenance of a healthy, vascularised and innervated dental pulp is necessary for a healthy tooth and dental regeneration (Huang et al., 2018). In the dental pulp cavity, MSCs are known to dwell within peri-vascular microenvironments, termed niches (Kaukua et al., 2014; Shi and Gronthos, 2003; Sui et al., 2019), and other locations (Gronthos et al., 2000; Miura et al., 2003; Morsczeck et al., 2005; Seo et al., 2004). However, little is understood about exact localisations and signalling regulations of the niches (Bluteau et al., 2008). The role of specific local niches essential to regulate cell migration, differentiation and cell fate specification during developmental and reactional events of dentine is not well recognised (Ruch, 1985). Dental progenitors/MSCs are capable of differentiating into new odontoblast-like cells, which can form a dentine-like structure such as RD, for dentine repair after a dentine injury such as dental caries.

### Dental caries and its management

Dental caries, the most prevalent chronic infectious disease globally, is a biological irretrievable impairment of vulnerable dental hard tissues due to acids produced by bacterial glycolysis of dietary carbohydrates (Baker et al., 2021). The WHO has defined the early childhood caries as a worldwide problem, with a prevalence between 60 % and 90 %. In addition, more than 90 % of all adults have experienced this disease (Bernabe et al., 2020; Griffin et al., 2008; Kazeminia et al., 2020). Tooth decay leads to dental pulpal infection, necrosis, loss of tooth function and vitality as well as eventual loss of the tooth. Various restorative materials have successfully been used to fill and replace injured or diseased dental tissues (Wang et al., 2020b). However, after restorative treatment, about 50 % of cases demand revision in 5-10 years (Burke and Lucarotti, 2009; Chen et al., 2020). In addition, any traditional artificial restorative material might fail due to inappropriate physical, biocompatible and mechanical properties (Goldberg and Smith, 2004; Tziafas et al., 2000; Yang et al., 2020a). If the material pulls away from the cavity wall, a microleakage would form between the dentine layer and dental materials, causing secondary or recurrent caries (Askar et al., 2021; Goldberg and Smith, 2004; Tziafas et al., 2000). Therefore, despite several advances in dental restorative materials, it is required for new therapeutic restorative methods in dentistry to support a healthy dentition. Therapies using stem cells such as dental pulp MSCs, cell/tissue engineering and other biomaterial components have successfully been reported for replacing or regenerating destroyed and injured dental tissues (Han et al., 2021; Saoud et al., 2016). For instance, Vidovic et al. (2017) showed that when pulpectomy is performed in animal models, a group of dental pulp progenitor cells/MSCs can migrate to the injured areas, differentiating into odontoblast-like cells, forming an RD. Consequently, the growth factor BMP2 enhances dental pulp cell differentiation into odontoblast-like cells, which synthesise and secrete dental ECMs, forming an RD in the injured areas (Nakashima, 2005; Ni et al., 2018). Besides BMP/TGF-β signalling, recent studies have demonstrated that Wnt/β-catenin signalling induces progenitor cell/MSC growth and differentiation, promoting RD formation (Neves and Sharpe 2018; Zaugg et al., 2020). Furthermore, Han et al. (2021) reported that an artificial synthesised peptide, termed TVH-19, promotes human dental pulp cell differentiation and induces tertiary dentine formation in a rat model.

### Dental pulp MSCs

Stem cells are characterised by both self-renewal and differentiation potential. The self-renewal of stem cells can occur by symmetric cell divisions, generating two daughter cells with the same fate, or asymmetric cell divisions, where one daughter cell is identical to the mother cell, while the other develops into a different cell type (Götz and Huttner, 2005).

Stem cells are classified as ESCs, iPSCs and ASCs. ESCs originate from the inner cell mass of the blastocyst prior to implantation. ESCs possess unlimited self-renewal potential and can generate all the body cell types. iPSCs, generated by inducing the expression of defined transcription factors in somatic cells, are pluripotent and can differentiate towards all cell types in given microenvironments. ASCs reside within different tissues such as the BM. Unlike ESCs and iPSCs, ASCs are limited in their potential to the cell types of the tissue they inhabit. Although stem cells normally remain in a quiescent, nondividing state, ASCs can proliferate and differentiate to replace damaged cells within their tissues and accelerate tissue healing following an injury (Cable et al., 2020; Pittenger et al., 1999; Yamanaka, 2020).

The BM contains numerous different cell types arising from HSCs, non-haematopoietic MSCs and other cell types, which are interconnected by a vascular and innervated network within the cavities of the BM. HSCs have the ability of self-renewal and differentiation into various cell types including erythrocytes, megakaryocytes, platelets, granulocytes, lymphocytes, osteoclasts, and dendritic cells (cells of the erythroid/myeloid lineages) and others. Subsequently, HSCs migrate to other haematopoietic or lymphoid organs giving rise to B lymphocytes, T lymphocytes, macrophages, and others. MSCs produce osteoblasts (bone-forming cells), adipocytes (fat cells) and other cell types, while osteoclasts (bone-resorbing cells) share a monocytic origin with macrophages. MSCs display a variable self-renewal and differentiation potential (Friedenstein et al., 1970; Pittenger et al., 1999; Wilkinson et al., 2020). They have been widely characterised *in vitro* as expressing various markers such as STRO-1, CD146 or CD44 (Pittenger et al., 1999). STRO-1 is a cell surface marker of osteogenic precursors, CD146 and CD44 are pericyte and mesenchymal stem cell markers, respectively. MSCs have self-renewal ability and potentially differentiate into mesodermal lineages, therefore originating cartilage, bone, fat, skeletal muscle and connective tissues (Pittenger et al., 1999; Wang et al., 2020a). Endothelial progenitor/stem cells play a principal role in BM angiogenesis as they have clonogenic capability and can be mobilised into the peripheral blood system, differentiating into mature endothelial cells in newly formed blood vessels after tissue injury. Thus, endothelial stem cells derived from the BM represent a source for the body vasculogenesis and angiogenesis.

Dental pulp contains progenitor cells/MSCs able to differentiate into adipocytes, chondrocytes, odontoblasts, osteoblasts and other cell type in given environments. During dentineogenesis and tertiary dentine formation, dental pulp progenitors/MSCs are able to differentiate into odontoblast-like and odontoblastic cells under appropriate signals (Gronthos et al., 2000; Miura et al., 2003; Sui et al., 2019). In a tooth, some cells can be either transit-amplifying cells or progenitors and commit to terminal differentiation. These transit-amplifying cells and progenitors have a limited lifespan thus, they can only produce a tissue for a given time (Walker et al., 2019). By contrast, dental MSCs are self-renewing and able to generate any of the tissues for their entire life span. In the dental pulp, dental MSCs include DPSCs, SHED and SCAP (Fig. 1b) (Gronthos et al., 2000; Miura et al., 2003; Sonoyama et al., 2008).

DPSCs were first isolated from human permanent third molar teeth and are the most common source of dental MSCs (Gronthos et al., 2000). DPSCs lack unique markers, therefore generic MSC markers such as STRO-1, CD146, CD105 and CD44 are used for the identification and isolation of DPSCs (Pittenger et al., 1999; Wang et al., 2020a). DPSCs can differentiate into odontoblasts (Gronthos et al., 2000), osteoblasts (d’Aquino et al., 2009), chondrocytes (Waddington et al., 2009), adipocytes (Gronthos et al., 2002; Waddington et al., 2009), myoblasts (Pisciotta et al., 2015) and neurogenic cells (Martens et al., 2014) *in vitro* and *in vivo*.

SHEDs were isolated from deciduous teeth, have fibroblastic features and express MSC specific markers including CD45, CD90, CD106, CD146, CD166 and STRO-1 but not haematopoietic and endothelial markers such as CD34 and CD31 (Miura et al., 2003). SHEDs have a high proliferation rate and can differentiate into adipogenic, chondrogenic, myogenic, neurogenic, odontogenic and osteogenic cells *in vitro* as well as induce formation of dentine and bone *in vivo* (Miura et al., 2003). SHEDs, neural-crest-derived stem cells, also express neural cell markers such as nestin, beta III tubulin and GFAP as well as several pluripotent markers including Oct4 and Nanog (Chai et al., 2000; Miura et al., 2003; Yang et al., 2019; Yang et al., 2020b). SHEDs express more osteocyte markers such as ALP, collagen type I and Runx2 than do BM MSCs *in vitro*. SHEDs were transplanted into the subcutaneous tissue in immunodeficient mice and promoted bone repair through inhibition of osteoclast activity *in vivo* (Yamaza et al., 2010). They are also capable of differentiating into vascular endothelial cells and form functional blood vessels by up-regulation of MEK1/ERK signalling (Bento et al., 2013). Due to their deciduous teeth origin, SHEDs exhibit several features similar to DPSCs. However, their proliferation and differentiation capacity are higher than that of DPSCs and BM MSCs (Bluteau et al., 2008).

SCAPs isolated from apical papilla cells at the root apex of teeth, display high proliferation rates and demonstrate an increase of migratory and regenerative capacities compared with other dental MSCs (Sonoyama et al., 2008). SCAPs are easily obtainable from human third molars. As SCAPs can be derived from the primary teeth, they express primitive embryonic markers including Sox2, Oct3/4, Nanog and others (Lee and Seo, 2016; Sonoyama et al., 2008). Among these markers, CD146 and STRO-1 co-expression is related to early-MSC phenotype. Certainly, CD146^+^/STRO-1^+^ SCAPs show superior colony-forming efficiency, with increased cumulative doubling compared with their counterpart (Nada and El Backly, 2018). CD24, another marker of the pluripotent population is considered to be a representative surface marker for SCAPs due to its absence in other dental MSCs (Kang et al., 2019). It is worth noting that the expression of the three markers CD146, STRO-1, CD24 declines with cell passaging, supporting their correlation with superior stemness. SCAPs are optimised for osteogenesis and odontogenesis; regarding that, SCAPs are considered to be odontoblast precursors *in vivo* (Du et al., 2020; Nada and El Backly, 2018). However, SCAPs are multipotent and give rise to mesenchymal cell lineages such as adipocytes and chondrocytes (Yang et al., 2020b). Taken together, SCAPs will hopefully gain a significant role in tissue repair and regeneration.

Besides dental MSCs (Gronthos et al., 2000; Miura et al., 2003), other MSC populations have been isolated from human dental tissues including the periodontal ligament (Seo et al., 2004) and the dental follicle (Morsczeck et al., 2005). Progenitors/stem cells isolated from the oral cavity express a group of mesenchymal markers, such as CD29, CD73, CD90 and CD105, and embryonic markers, including Sox2, Nanog and Oct4, and can differentiate into multiple cell lineages (Miran et al., 2016). Noticeably, some dental stem cells demonstrate more embryonic-like characteristics than those of BM and umbilical cord stem cells (Miran et al., 2016; Sui et al., 2019). Oral cavity MSCs are an important and valuable resource for dental and medical clinical/therapeutic applications. However, little is known about how progenitor cells/MSCs differentiate into specific mature cells, such as osteoblasts and odontoblasts, as well as which niches promote such differentiation.

## SIBLINGS and DSPP

**Fig. 2.**
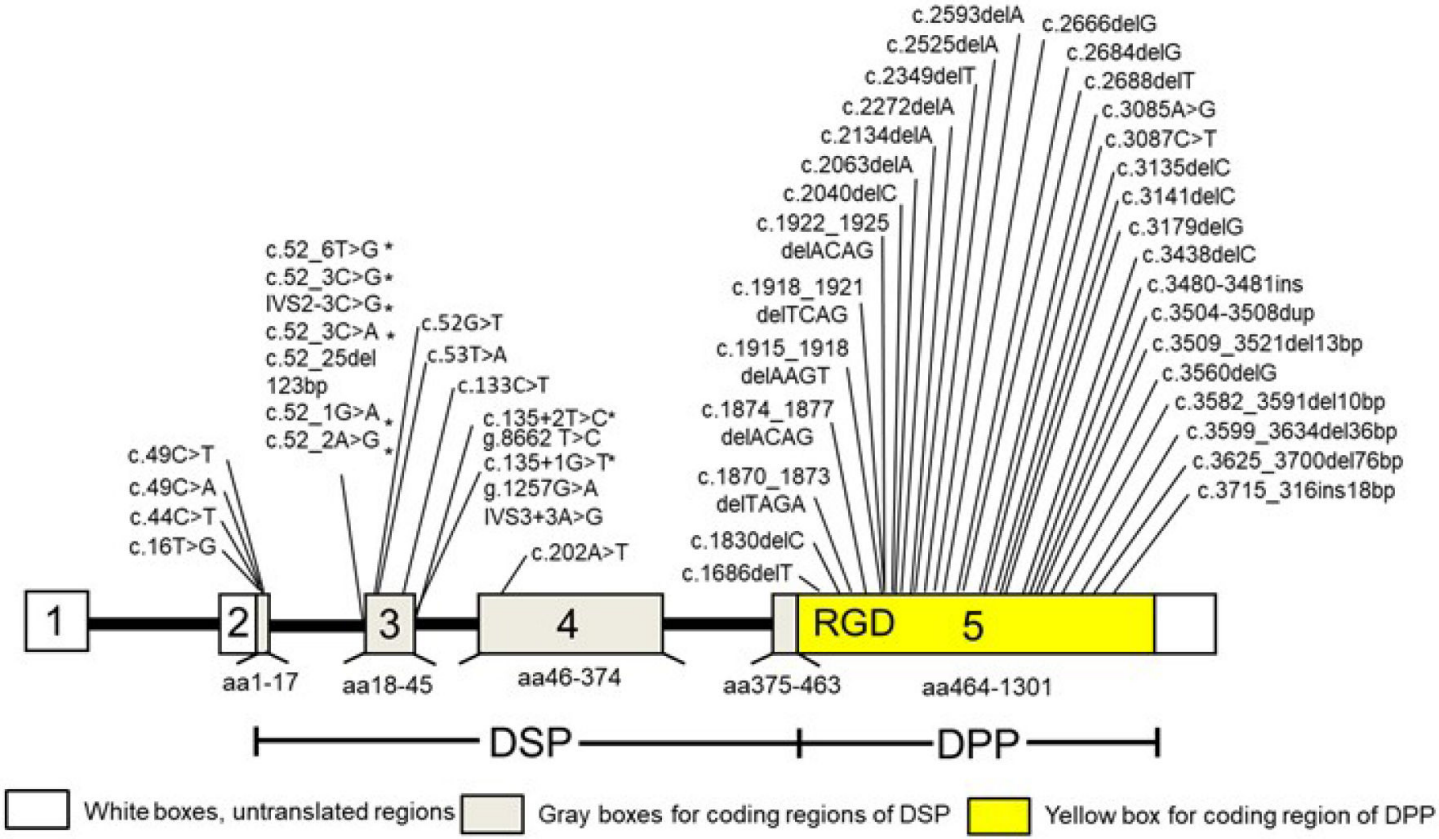
Diagram of *DSPP* mutations associated with genetic dentine diseases. The structure of the human *DSPP* is shown. Exons are shown as boxes numbered 1-5, with the amino acids (aa) encoded by each exon indicated below. Introns are represented by lines. Locations of *DSPP* mutations are indicated. White colour indicates the 5’ UTR, gray colour DSP sequences, yellow colour the DPP sequence. Asterisks show mutations affecting splice sites according to a splice-site recognition software (https://www.phenosystems.com/www/index.php). c. = cDNA; g. = genomic; del = deletion; dup = duplication; ins = insertion; IVS = intervening sequence.

**Fig. 3.**
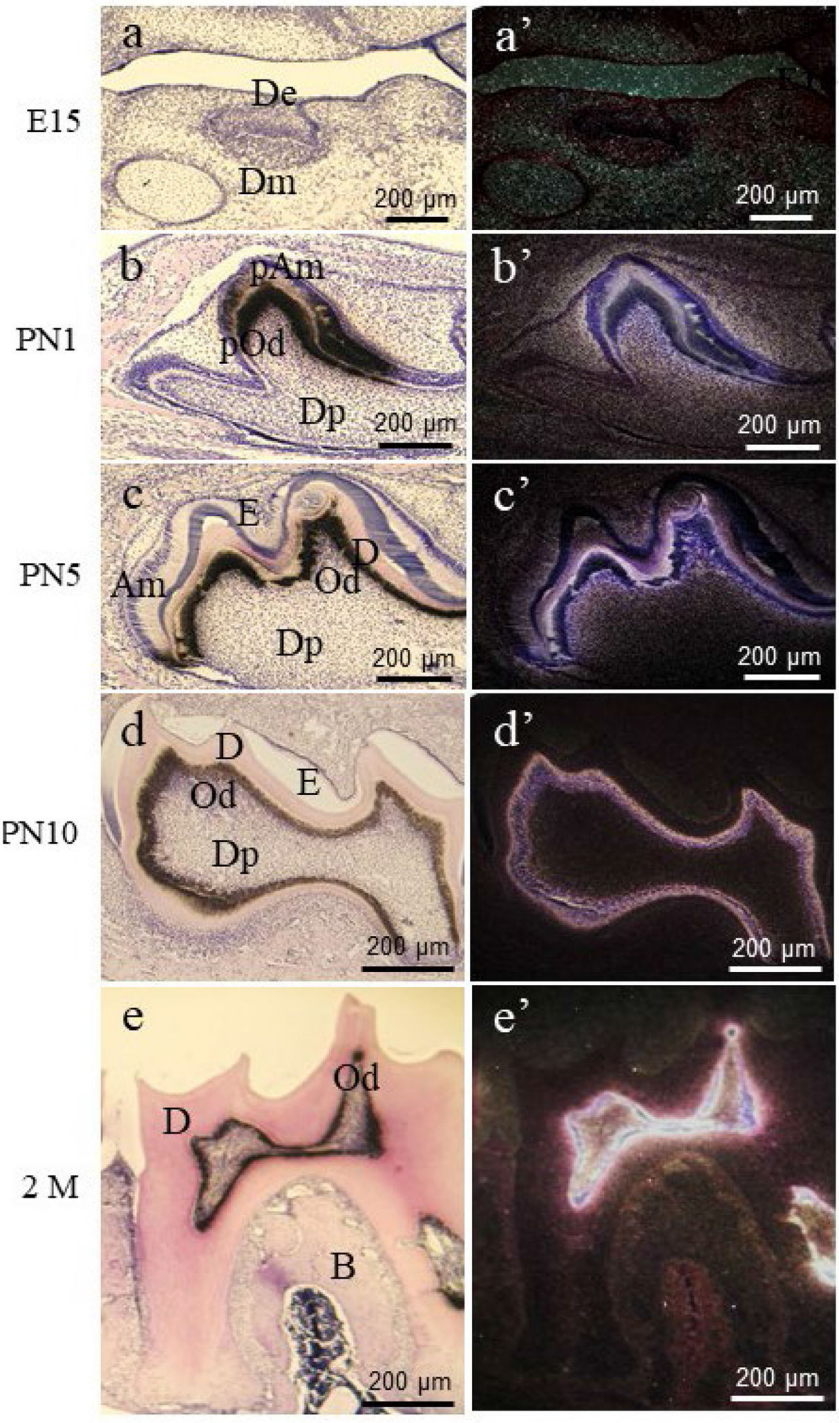
*DSPP* expression pattern in developing mouse teeth from embryonic day 15 to postnatal month 2. (**a’**) *DSPP* mRNA expression was not seen in the dental and osteogenic mesenchyme as well as the dental epithelium at embryonic day 15 (E15). (**b’**) At postnatal day 1 (PN1), *DSPP* expression was detected in pre-ameloblasts, pre-odontoblasts and weakly in the dental pulp. (**c’-e’**) *DSPP* expression was mostly restricted to odontoblasts from PN5 to 2 months (M) after birth while *DSPP* expression was barely seen in bones, (**a-e**) Brightfield images. Am, ameloblasts; B, bone; D, dentine; De, dental epithelium; Dm; dental mesenchyme, Dp, dental pulp; E, enamel; Od, odontoblasts; pAm, pre-ameloblasts; pOd, pre-odontoblasts

Niches can influence cell behaviour and fate (Méndez-Ferrer et al., 2020; Morrison and Spradling, 2008; Perry and Li, 2007). For instance, BM ECM influences osteoblast differentiation into osteocytes while dental pulp ECM governs dental progenitor cell/MSC differentiation into odontoblasts (Chen et al., 2005; Chen et al., 2007; Guo et al., 2009; Vijaykumar et al., 2020). Bone and dentine are highly mineralised tissues formed by osteoblasts and odontoblasts, which derive from mesenchymal cells. Both bone and dentine possess common characteristics and show similar features during mineralisation. During this process, odontoblasts and osteoblasts synthesise and secrete ECM proteins to form matrix-forming predentine and osteoid, respectively, which in turn are converted to bone and dentine. At the same time, the organic matrix of osteoid and predentine is composed of collagens and NCP proteins necessary for mineralisation of collagen fibres. The most common NCP proteins of bone and dentine include BSP, OPN, MEPE, DSPP and DMP1, belonging to the SIBLINGS family (Bellahcène et al., 2008; Fisher and Fedarko, 2003; MacDougall et al., 1997). SIBLING genes are located on chromosome 4q21 in humans and chromosome 5q in mice, sharing a similar exon structure. The presence of the RGD integrin-binding motifs enables them to trigger intracellular signals by initiating integrin-mediated signalling. Although bone and tooth show several common characteristics, the physical and biological functions of osteoblasts and odontoblasts exhibit several differences (Chen et al., 2005; Chen et al., 2009; Vijaykumar et al., 2020). The functions of the members of the SIBLINGS family in dentine and bone have been found through a linkage to human diseases and different genetic animal models. DSPP, ~ 143 kDa, is the largest of the SIBLING proteins, with 1,301 amino acids in humans and plays essential roles in dentineogenesis (de La Dure-Molla et al., 2015). *DSPP* contains 4 introns and 5 exons (Fig. 2) (MacDougall et al.,1997). Unlike other SIBLING protein family members, DSPP spatial-temporal expression is mainly seen in pre-ameloblasts and odontoblasts during tooth development and formation (Chen et al., 2009; D’Souza et al., 1997) and weakly detected in osteoblasts and non-mineralised tissues (Fig. 3) (Chaplet et al., 2006; Qin et al., 2002). For example, DSPP protein expression in odontoblasts and dentine is about 400-fold higher than that in osteoblasts and bone (Qin et al., 2002). *DSPP* is transcribed from a single gene (MacDouall et al., 1997) but full-length DSPP has hardly been found in cells or tissues, whereas its cleavage products, DSP and DPP in mice, rats and humans as well as DSP, DGP and DPP in pigs are the most abundant NCPs in dentine and odontoblasts (Qin et al., 2001; Yamakoshi et al., 2005; Yuan et al., 2012).

DSP is composed of partial exon 2, exon 3, exon 4 and partial NH_2_-terminal region of exon 5 of *DSPP*, while DPP consists of most *DSPP* exon 5 (Fig. 2). DSPP is first processed into DSP/DGP and DPP (also termed dentine PP) by BMP1, TLR metalloproteinases and astacin proteases (Marschall and Fisher, 2010; Steiglitz et al., 2004; Tsuchiya et al., 2011). Then, DSP is further catalysed into small active molecules by MMP-2, −9 and −20 to expose cryptic binding sites into active molecules (Yamakoshi et al., 2006; Yuan et al., 2017). Mutations of the cleavage site between DSP and DPP regions result in DGI phenotypes in mice, indicating that DSPP needs to be cleaved into its active fragments, DSP and DPP (Zhu et al., 2012).

The porcine DGP has an 81 amino acid segment of DSPP (Ser^392^ to Gly^472^) located between DSP and DPP fragments. DGP contains 4 phosphorylated serine residues (Ser^453^, Ser^455^, Ser^457^ and Ser^462^) and 1 glycosylated asparagine (Asn^397^). DGP molecular weight is a 19 kDa in SDS-APGE gel by Coomassie Brilliant Blue staining, that is decreased to 16 kDa by glycopeptidase A digestion. The porcine DGP has the same number (12 each) of positively charged (Arg and Lys) and negatively charged (Asp and Glu) residues. This pig DGP contains abundant Ser (12) and Gly (13). Lacking post-translational modifications, DGP has a calculated isoelectric point of 6.7. Due to containing 4 phosphorylated Ser and sialic acids, the modified DGP has an increased affinity for HA, which most likely facilitates the binding to dentine crystals. The identity of the porcine DGP amino acid sequence (NP_99842.1) is conserved, with 58 (81 %) conserved amino acids in humans (F42472.1), 40 (49 %) in rats (L79813.1) and 38 (47 %) in mice (C12787.1) (Yamakoshi et al., 2005). How DSPP is catalysed into the porcine DGP by proteinases and DGP functions during dentineogenesis are yet to be determined.

**Fig. 4.**
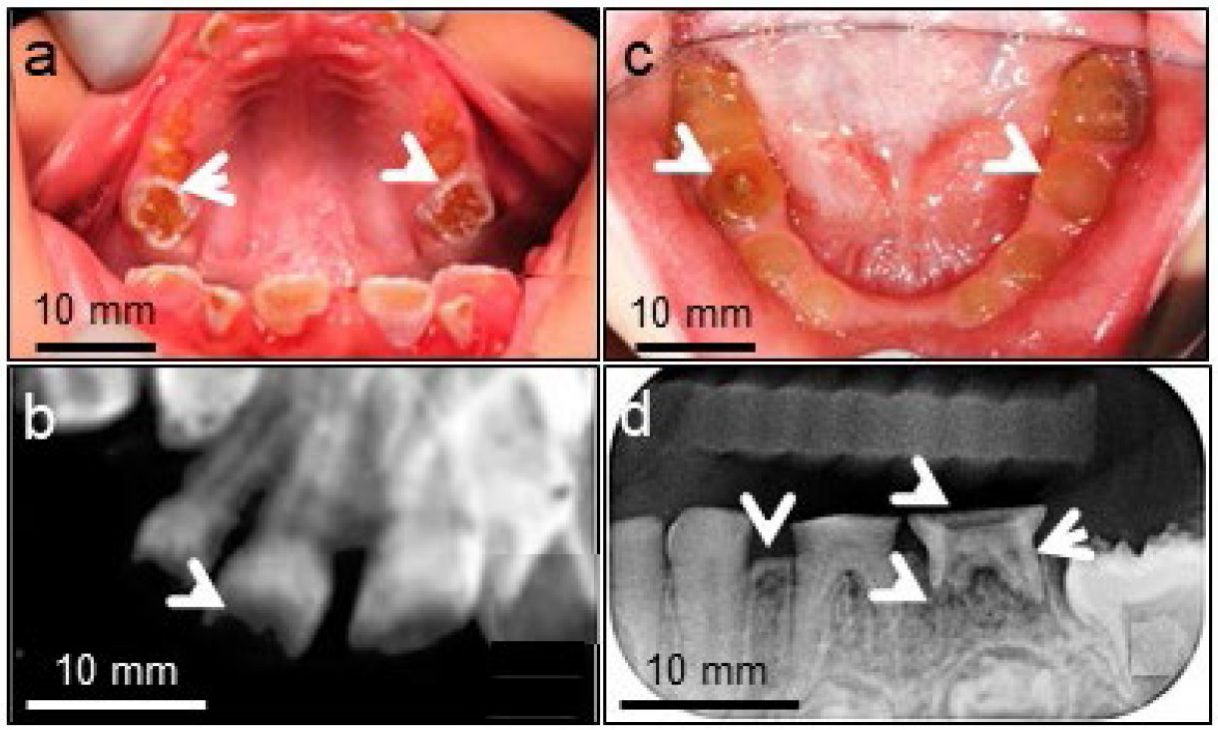
Clinical photographs and radiographs from DGI-II patients. Clinical photograph of a 7 year-old boy showing (**a**) severe attritions (arrowheads). (**b**) Radiograph indicates severe enamel loss with decreased pulp space and reduced dental mineral density. (**c**) Intraoral photographs of a 5 year-old girl exhibiting severe attrition of the primary dentition to the gingiva level and teeth with yellow-brown colour and a translucent appearance (arrowheads). (**d**) Radiograph shows that dentine was thin, with severe occlusal attrition and periapical abscess (arrowheads).

DSP and DPP play unique biological roles during tooth development (Paine et al., 2005; Suzuki et al., 2009). DSP or DPP mutations in humans are associated with DGI-II (OMIM 125490) and DGI-III (OMIM 125500) as well as DD-II (OMIM 125420) and DD-I (MIM 125400) (Fig. 2, Table 1,2). Those hereditary dentine disorders are the most common dentine genetic diseases. Estimated incidences of DGI in humans is 1/6,000-8,000, while DD is 1/100,000 (Witkop, 1975). DGI-II is characterised by pulpal calcification, opalescent discoloured dentition and bulbous crown shape as well as impaired odontoblast cell differentiation and delayed conversion of predentine to dentine (Fig. 4). DGI-III was originally regarded as a Brandywine isolate (Witkop, 1975) and a severe form of DGI-II with multiple dental pulp exposures and shell-like teeth. DD-II is similar to DGI-II in the deciduous dentition, but tooth discolouration is minimal and dental pulp cavities are thistle-tube shaped with pulp stones in the permanent dentition. In DD-I, teeth are normal in shape and form, as well as consistent in the deciduous and permanent dentitions. In some cases, colour of the teeth may exhibit a slightly amber discolouration. However, the roots are short, and the pulp obliteration causes a crescent-shaped pulpal remnant in the permanent dentition and a total pulpal obliteration in the deciduous dentition. Using mouse models, it was confirmed that *Dspp* is required for dentineogenesis, as homogenous null mice (*Dspp*^−/−^) show tooth deficiency similar to those seen in patients suffering from DGI and DD, with enlarged pulp cavities, a wide predentine zone, reduced dentine volume, hypomineralisation and dental pulp exposure (Fig. 4) (de La Dure-Molla et al., 2015; Sreenath et al., 2003).

SIBLING-RGD motifs are capable of binding to cell surface integrins in normal tissues and enhance cell adhesion, spreading, motility, proliferation, differentiation and survival *via* up-regulating kinase cascades and transcription factors. Also, the biological functions of SIBLINGS are regulated by proteolytic processing to uncover cryptic binding sites and expose functional domains, thus modulating cell adhesion and activity. For instance, OPN protein interacts with various integrins, such as αvβ3, αvβ5, αvβ1, α4β1, α8β1, α9β1 and CD44 splice variants (Bellahcène et al., 2008; Marschall and Fisher, 2008). OPN by thrombin cleavage separates the CD44^−^ and integrin-binding domains, which in some cases promote adhesion over cell migration. Another example is the thrombin-cleaved NH_2_-terminal OPN segment that interacts with αvβ3 and αvβ5 integrins *via* the RGD motif (Bellahcène et al., 2008; Furger et al., 2003) or with α4β1 and α9β1 integrins *via* the cryptic SVVYGLR sequence (Rangaswami et al., 2006) and promotes cell adhesion and migration. The COOH-terminal region of OPN interacts with CD44 variant 6 (CD44v6) and/or variant 3 (CD44v3) by a heparin bridge (Teramoto et al., 2005). In addition, OPN is also catalysed by MMP-3 and MMP-7 and the cleaved OPN domains promote cell adhesion and migration *in vitro* by activating β1-containing integrins (Agnihotri et al., 2001). OPN is also a substrate for plasma transglutaminase factor IIIa and liver transglutaminase (Prince et al., 1991) and enhances cell adhesion, spreading and migration (Higashikawa et al., 2007). The RGD domain of DMP1 only binds to αvβ3, while BSP-RGD motif not only interacts with αvβ3, but also with αvβ5 and enhances cell adhesion and migration (Marschall and Fisher, 2008). DMP1 is a substrate of BMP1 and BMP1-generated DMP1 fragments have similar binding efficiency to the intact DMP1 protein in cell attachment and migration (Marschall and Fisher, 2008; Steiglitz et al., 2004).

### DPP

**Fig. 5.**
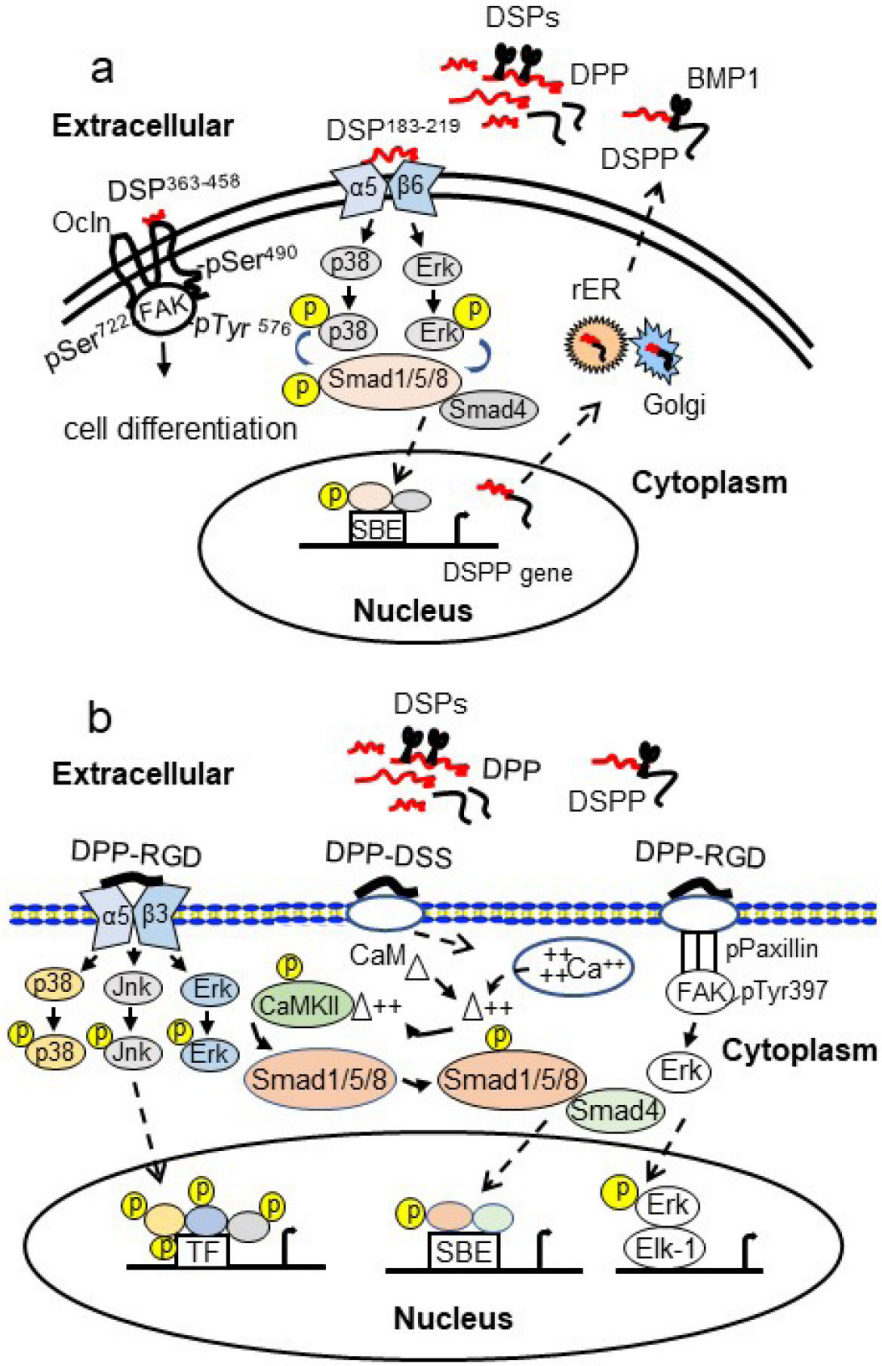
Hypothetical model of DSPP signalling during dentineogenesis and dentine regeneration. (**a**) DSP^183-219^ binds to integrin β6 and forms a complex, activating phosphorylation of p38, Erk1/2 and Smad1/5/8. Phosphorylated Smad1/5/8 interact with Smad4. The complex is translocated into the nucleus. Phosphorylated Smad1/5/8 in coordination with Smad4 bind to SBEs in the DSPP regulatory region and activate *DSPP* transcription. On the other hand, DSP^363-458^ as a ligand interacts with the extracellular loop2 of Ocln^194-241^, stimulating Ocln phosphorylation at Ser^490^. Furthermore, the DSP-Ocln complex stimulates FAK phosphorylation at Ser^722^ and Tyr^576^ and then induces dental mesenchymal cell differentiation and mineralisation. (**b**) DPP binds to integrin α5β3 through its RGD domain. DPP-α5β3 activates MAPK signal pathway and up-regulates gene expression and cell differentiation. The RGD domain of DPP phosphorylates paxillin and FAK at Tyr^397^. Phosphorylated FAK activates Erk and phosphorylated Erk is translocated into the nucleus and activates transcription factor Elk-1 and downstream gene expression. In addition, the DSS repeat region of DPP mediates intracellular calcium store flux and triggers CaMKII activation, resulting in Smad1/5/8 signalling cascade. DSS, Asp-Ser-Ser; RGD, Arg-Gly-Asp.

DPP contains an RGD domain at the NH_2_-terminal site, acting as a ligand and binding to integrin αVβ3. DPP-RGD/integrin-αVβ3 complex activates intracellular signalling pathways through up-regulating *MAPK*, including *SAPK/JNK, ERK1/2* and *p38* in human and mouse cells. Consequently, this complex up-regulates bone/dentine-related gene expression such as *RUNX2, OSX, ALP, OCN* and *BSP* in human and mouse cells as well as promotes cell differentiation and mineralisation in hBMSC, mouse osteoblastic cells (MC3T3-E1) and mouse fibroblastic (NIH3T3) cells (Jadlowiec et al., 2004; 2006). In addition, DPP-RGD induces phosphorylation of paxillin, FAK and of the transcription factor Elk-1 and up-regulates downstream gene transcription in mouse embryonic mesenchymal (C3H10T1/2) and primary dental pulp cells (Eapen et al., 2012) (Fig. 5). The flanking regions of the RGD motif influence binding of RGD to specific integrins and enhance cell adhesion and migration (Marschall and Fisher, 2008; Suzuki et al., 2014). However, unlike other SIBLING family members, 17 out of 37 DSPP genes from 37 species tested do not contain the RGD motif of DPP, indicating that the RGD domain within the DPP may be rudimental (Suzuki et al., 2016).

In addition to these common domains, only the DPP domain of DSPP contains abundant Ser-Asp or Asp-Ser repeat regions, which are the most phosphorylated regions of SIBLING protein and one of the most acidic proteins in numerous species such as human, rat and mouse (Jonsson et al., 1978; Suzuki et al., 2016). DPP binds to calcium ion and collagen type I, acting as an inductor of mineralisation in ECMs and inducing HA deposition and growth of vertebrate bones and teeth (He et al., 2005). DPP can interact with the cellular membrane (annexin 2 and 6) and facilitates calcium influx into cells (Alvares et al., 2013) while functioning as a cell-penetrating peptide promoting cellular uptake of components attached to it and releasing different cargos intracellularly (Figueiredo et al., 2019; Ravindran et al., 2013). Additionally, DPP-DSS (Asp-Ser-Ser) repeat regions can facilitate intracellular Ca^2+^ release. This calcium flux promotes the activation of Ca^2+^ CaMKII. Activated CaMKII enhances the phosphorylation of the transcription factors Smad1/5/8 and phosphorylated Smad1/5/8 proteins are translocated to the nucleus and up-regulate Smad1/5/8 downstream gene expression as well as promote cell differentiation in murine pluripotent stem cells (C3H10T12) and hBMSCs (Eapen et al., 2013) (Fig. 5). Eapen et al. (2013) showed that the length of the Ser-Asp and/or Asp-Ser repeat regions varies among species but is not correlated with dentine hardness (Suzuki et al., 2016).

To analyse the relationship between length variations in Ser-Asp/Asp-Ser repeat regions and the role of DPP in matrix mineralisation, different lengths of the Ser-Asp/Asp-Ser repeat regions have been generated (Kobuke et al., 2015). Recombinant mouse Dpp deleted 63.5 Ser-Asp repeat regions, accounting for 36.5 % of the length of the Ser-Asp repeat region, were generated and these peptides were able to induce calcium-phosphate precipitation similarly to the full length Dpp at the same concentration. In contrast, the inverted Dpp deleted 63.5 Ser-Asp repeat regions had no effect on the induction of calcium phosphate precipitation (Kobuke et al., 2015). The 8-repeat copy of Asp-Ser-Ser residues facilitates calcium-phosphate precipitation and HA crystal growth, promoting the remineralisation of demineralised human enamel and dentine tubule occlusion (Hsu et al., 2011). Dpp-mimetic peptide molecules upregulate the expression of bone/dentine-related genes including *RUNX2, ALP, DMP1, OCN* and collagen type I in human osteosarcoma (Saos-2) cells as well as promote cell differentiation (Gulseren et al., 2019). The biological function of Dpp was narrowed-down to 3 Asp-Ser-Ser repeat peptides that are able to facilitate calcium-phosphate deposition on the human enamel surface and crystallographic structure of calcium-phosphate crystals *in vitro* (Chung et al., 2012).

For the *in vivo* study of the role of DPP, mice overexpressing *Dpp* transgenic gene driven by *Col1α1* promoter (*Dpp-Col1α1* Tg) were crossed-bred with *Dspp* KO (*Dspp*^−/−^) mice to generate *Dspp* KO/*Dpp Col1α1* Tg mice (Zhang et al., 2018). *Dspp* KO/*Dpp-Col1α1* Tg mice had an increase in dentine thickness and restored dentine mineral density compared with *Dspp* KO mice. Histochemistry showed that abnormal widening of the predentine was narrower in *Dspp* KO/*Dpp-Col1α1* Tg mice. Scanning electron microscopy analysis demonstrated that the structure of dentinal tubules in *Dspp* KO/*Dpp-Col1α1* Tg mice was better organised than that of *Dspp* KO mice. Dentine mineral deposition rate in *Dspp* KO/*Dpp-Col1α1* Tg mice was significantly enriched compared to that of *Dspp* KO mice as analysed by double fluorochrome labelling. The overexpression of Dpp partially rescued the dentine deficiency in *Dspp* KO mice, indicating that Dpp may facilitate dentine development during dentineogenesis. In contrast, the body weight of *Dpp-Col1α1* Tg mice was lower compared to that of wild type mice. Moreover, both short and long bones were shorter in *Dpp-Col1α1* Tg mice compared to that of wild type mice. *Dpp-Col1α1* Tg mice presented reduced trabecular bone formation and exhibited narrow proliferative and chondroblast layers in long bones. Histochemistry analysis demonstrated that the proliferative zone of long bones in *Dpp-Col1α1* Tg mice was characterised by reduced cell proliferation and increased gene expression of chondroblast differentiation markers such as type II collagen (a marker of proliferating chondrocytes), type X collagen (a marker of hypertrophic chondrocytes) and proteoglycan, but there were no obvious defects in chondrocyte differentiation (Zhang et al., 2016). Transgenic mice of an overexpression of *Dpp* driven by the mouse *Amg* promoter (*Dpp-Amg* Tg) were generated. *Dpp-Amg* Tg mice showed a pitted and chalky enamel with nonuniform thickness that tended to wear more easily. In mice, *Dpp-Amg* transgene results in disruptions of the prismatic enamel structure and weakened enamel with uneven thickness (Paine et al., 2005; White et al., 2007). The reasons for the different effects of Dpp on different tissue development and formation remain unclear. A reason might be that the biological mechanisms of Dpp are cell- and tissue-specific. Spatial-temporal expression of *Dspp* is detected in preodontoblasts and preameloblasts at early stages of tooth development. During mouse tooth formation at postnatal stages, *Dspp* expression is barely detected in ameloblasts, but continuously seen in odontoblasts, predentine and dentine, maintaining odontoblast and dentine metabolism and homeostasis (Fig. 3) (Chen et al., 2009; D’Souza et al., 1997). However, *Dspp* is weakly expressed in osteoblasts, chondrocytes and bones (Chen et al., 2009; Qin et al., 2002). This suggests that a dose-dependent tuning of *Dspp* expression plays important roles in cell- and tissue-biological activity and behaviour. For instance, *Runx2* is a key factor necessary for osteoblast differentiation and bone formation (Ducy et al., 1997). *RUNX2* mutations in humans are related to CCD, with affected subjects displaying short stature, late closure of fontanels and sutures, aplasia of clavicles, hypertelorism, low nasal bridge and dental defects including tooth hypoplasia supernumerary teeth and abnormal tooth eruption (Lee et al., 1997). *Runx2* is expressed by dental mesenchymal cells at the early stages and downregulated in odontoblastic cells at the later stages during odontogenesis (Chen et al., 2009). *Runx2* stimulates *Dspp* expression in mouse preodontoblastic cell lines but represses its expression in mouse odontoblastic cells (Chen et al., 2005). *Runx2*^−/−^ mice present impairment of tooth formation, with progression only to the cap/early bell stages of tooth development. The teeth in *Runx2*^−/−^ mice are misshapen, severely hypoplastic and lack odontoblast and ameloblast differentiation, while exhibiting loss of normal dentine and enamel matrices (D’Souza et al., 1999). In contrast, in *Runx2* Tg mice, odontoblasts lose their normal columnar shape and dentine is surrounded by odontoblasts that are flat or/and cuboid in shape. In *Runx2* Tg mice, dentine is thin and retains lacunae, which display osteoblast and bone-canaliculi-like structures. Structure of dentinal tubules and pre-dentine is invisible. Moreover, collagen type I expression is decreased and *Dspp* expression is undetectable (Miyazaki et al., 2008). Therefore, *Runx2* function is related to cell- and tissue-type-specific or dependent on the stages of cytodifferentiation during tissue development.

### DSP

DSP lacks an RGD domain and Ser-Asp/Asp-Ser repeat regions (MacDougall et al 1997; Suzuki et al., 2016). Many DSPP mutations occur in the DSP region (Fig. 2, Tables 1,2). DSP and peptides derived from it are able to regulate gene expression, protein phosphorylation and induce dental primary/stem cell differentiation (Lee et al., 2012; Ozer et al., 2013).

The starting site of DSP contains the signal peptides, which are required for intracellularly trafficking of DSPP from the rER to the ECM. Point mutations of the signal peptides such as Tyr 6 to Asp, Ala 15 to Val, Pro 17 to Leu and Val 18 to Asp together with frameshift mutations resulting in longer mutant hydrophobic domains of DSPP are associated with DD-II, DGI-II and DGI-III (Fig. 2, Table 1,2). In a mouse model, an amino acid on Pro 19 of the signal peptides of Dspp was substituted by an amino acid on Leu 19 (Liang et al., 2019). The mutant mice Dspp^P19L/P19L^ displayed symptoms similar to human DGI-II and DGI-III, showing enlarged dental pulp chambers in mutant young mice and smaller dental pulp chambers in older mutant mice. These mutant mice exhibited an increase in enamel attrition and an undue deposition of peritubular dentin. Dspp^P19L/P19L^ mice presented a decrease in Dspp expression in odontoblasts as compared to the wild type mice. The secretion of the mutated Dspp was impaired and the mutant Dspp protein accumulated within the rER. The traffic mechanisms of Dspp protein from rER to ECM related to the mutations in the signal peptides associated with DGI and DD are not completely known. Recently, Yin et al. (2018) found that Surf4 (also named Erv29p) is the cargo receptor, which has a high affinity for binding the triple amino acids, IPV, within the signal peptides of DSPP but weakly binds the mutant amino acids of the signal peptides. The wild type DSPP is transported from the rER lumen to the ECM. Specific alterations in a single amino acid of the tripeptide of Dspp result in inadequate aggregate formation of Dspp within the rER and failure to efficiently transport Dspp out of the rER. The mutant signal peptide(s) of Dspp protein accumulate in the rER lumen, forming damaging aggregates and degradation by proteinases within rER (Yin et al., 2018).

DSP is an ECM protein that activates intracellular signalling pathways when dental cells are treated with it (Lee et al., 2012; Ozer et al., 2013). How DSP domain and its cleaved products facilitate intracellular signalling is unknown. Dsp protein was used as a bait for seeking its partner(s) through screening a dental cell protein library and it was found that Dsp acts as a ligand and interacts with 4 cellular membrane proteins including Ocln, integrin β6, CD105 (endoglin) and collagen type IV (Li et al., 2017; Wan et al., 2016). Dsp^183-219^ 36 amino acids are sufficient for interacting with the cellular membrane receptor integrin β6. Dsp-integrin β6 complex stimulates p38 and Erk1/2 phosphorylation and phosphorylated transcription factors Smad1/5/8 (pSmad1/5/8). pSmad1/5/8 interacts with Smad4 and both are translocated into the nucleus, bind to *Dspp* regulatory region, upregulate *Dspp* transcription and have a positive feedback on *Dspp* expression and odontoblast cell homeostasis. Also, Dsp^183-219^ peptide promotes dental cell spreading, migration, proliferation and differentiation. On the other hand, the COOH-terminal domain of Dsp^363-458^ binds to the second loop^194-241^ of Ocln, which is an integral membrane protein (Cong and Kong, 2020). Dsp domain phosphorylates Ocln on Ser^490^ and FAK on Ser^722^ and Tyr^576^ through binding of Ocln to FAK. Dsp^363-458^ facilitates mouse dental papilla mesenchymal and human dental pulp stem cell differentiation and mineralisation. Furthermore, in an *in vivo* study, Dsp^363-458^ was mixed with agarose beads (Dsp-beads) and the Dsp-beads compound was implanted into mouse dental pulp chambers. The histological analysis showed that in Dsp-beads-treated mice, dental pulp mesenchymal cell proliferation and cell differentiation were significantly improved around the Dsp-beads compound compared to that of the control mice. The dental pulp mesenchymal cells in the Dsp-beads-treated groups secreted dental ECMs and formed a layer between the dental pulp chamber and resin. More interestingly, there were a lot of newly formed blood vessels and less inflammatory cells around the Dsp-beads, along with the dental pulp mesenchymal cells and blood vessels, which migrated into the Dsp-beads. This study indicated that the Dsp^363-458^ is capable of inducing dental mesenchymal cell proliferation, cell differentiation and vasculogenesis (Fig. 5) (Li et al., 2017).

For the *in vivo* study of the biological role of DSP, overexpression of *Dsp* Tg mice driven by the mouse *Dspp* promoter (*Dsp-Dspp* Tg) was generated (Suzuki et al., 2009). *Dsp-Dspp* Tg mice were crossed-bred with the *Dspp*^−/−^ mice. *Dspp*^−/−^/*Dsp-Dspp* Tg mice resulted in partial rescue of restored predentine width, decrease of frequent dental pulp chamber exposure and partial recovery in dentine volume compared to *Dspp* KO mice. However, no rescue of dentine mineral density was observed in these *Dspp*^−/−^/*Dsp-Dspp* Tg mice. This study implies that Dsp is related to the initiation of dentine mineralisation. In addition, overexpression of the *Dsp* Tg mice driven by the mouse *Amg* promoter (*Dsp-Amg* Tg) causes significantly and uniformly increased enamel hardness and an increased rate of enamel mineralisation but did not significantly alter enamel morphology. These studies demonstrated that Dsp significantly contributes to the physical properties of the dentine-enamel junction and facilitates enamel formation (Paine et al., 2005; White et al., 2007). In contrast, *Dsp* driven by *Col1α1* promoter (*Dsp-Col1α1* Tg) Tg mice were crossed-bred with *Dspp* KO mice to generate *Dspp* KO/*Dsp-Col1α1* Tg mice (Gibson et al., 2013). Unexpectedly, dentine of *Dspp* KO/*Dsp-Col1α1* Tg mice was much thinner, more poorly mineralised and remarkably disorganised than that of *Dspp*^−/−^ mice. *Dspp* KO/*Dsp-Col1α1* Tg mice displayed more severe dentine defects than *Dspp*^−/−^ mice. Furthermore, *Dspp* KO/*Dsp-Col1α1* Tg mice resulted in severely worse periodontal defects than that of *Dspp* KO mice and a greater decrease of alveolar bone, more remarkably altered canalicular structures around the osteocytes, less cementum, more radical migration of the epithelial attachment towards the apical direction and more severe inflammation in molar furcation region than that of *Dspp* KO mice (Gibson et al., 2014). Overall, this suggests that the Dsp mediates an inhibitory role in periodontium formation. The different Dsp effects on hard tissue development and formation may rely on the control of given tissue gene promoters.

## Conclusions and future perspectives

The present review provides a brief overview of DSPP expression, proteolysis, pathophysiology and biological functions of the cleaved products, DSP/DGP and DPP, based on the recent literature. Dentine is a highly mineralised tissue and derives from odontoblasts. When dentine is injured, such as in cases of pulpotomy and dental caries, dental pulp progenitors/MSCs can migrate to the injured areas and differentiate into odontoblast-like cells (Vidovic et al., 2017). The differentiation of the dental pulp progenitors/MSCs is controlled at the given niches (Méndez-Ferrer et al., 2020; Morrison and Spradling, 2008). During dentineogenesis, odontoblasts synthesise and secrete dental ECMs, which bind to calcium-phosphate, finally forming predentine and dentine. Dental ECMs are composed of collagens and NCPs (MacDougall et al., 1997). Among NCPs, DSPP expression is highly visible in odontoblasts and dentine (Fig. 3) (Chen et al. 2009; D’Souza et al., 1997). DSPP is catalysed into DSP/DGP, DPP by BMP1 and TLR proteinases (Marschall and Fisher, 2010; Yamakoshi et al., 2006). Mutations of DSP and DPP domains are associated with DD-I, DD-II, DGI-II, DGI-III and the most common genetic dentine diseases (Fig. 2, Table 1,2). DSP and DPP play unique roles during odontogenesis. DSP promotes the initial effect on early dentine development while DPP is related to HA crystal growth and mineralisation (Suzuki et al., 2016). DSP is a ligand and facilitates intracellular signalling *via* its cellular membrane receptors, integrin β6 and Ocln as well as induces dental pulp/MSC cell differentiation and mineralisation. Dsp^183-219^-β6 signal up-regulates *Dspp* expression, dental cell proliferation and differentiation *via* p-p38-pErk-Smad1/5/8 signal pathways, while Dsp^363-458^-Ocln complex promotes dental mesenchymal cell/MSC differentiation and biomineralisation through FAK cascades (Fig.5a). Overexpression of Dsp partially rescues dentine defects in *Dspp* KO mice (Suzuki et al., 2009). In addition, DPP-RGD activates downstream gene expression and cell differentiation through integrin-MAPKs and paxillin-FAK signal pathways. Moreover, DPP contains Ser-Asp/Asp-Ser repeat regions, which mediate intracellular calcium store flux and trigger CaMKII-Smad1/5/8 activations, facilitating cell differentiation and mineralisation (Fig.5b). Dpp overexpression partially rescues dentine defects in *Dspp* KO mice (Zhang et al., 2018). Nevertheless, overexpression of *Dsp* or *Dpp* driven by the given gene promoter(s) results in impairment of certain tissues’ development (Gibson et al., 2014; White et al., 2007). How DSP and DPP play dual roles in different tissues is not completely understood and needs to be further studied. Although biological roles of DSPP have made the advanced achievements in odontoblast differentiation and mineralisation during tooth development, mechanisms of DSPP during tooth development and formation remain still unknown. For instance, where cleavages of DSPP occur in cytoplasm and/or ECMs needs to be further investigated. Differences of three-dimensional structures between wild type and mutant DSPP and its cleaved products have not been described and need to be studied. Control of the spatial-temporal cell- and tissue-specific expression of *DSPP* is not completely understood although *DSPP* expression is controlled by several growth factors, transcriptional factors and materials (Chen et al., 2008; Suzuki et al., 2016). However, understanding the mechanisms of *DSPP* spatial-temporal expression in odontoblastic cells at different stages during tooth formation and progenitor cell/MSC differentiation to odontoblasts may be a potential novel avenue during dentine development and regeneration.

## Figures and Tables

**Table 1.** Summary of *DSP* mutations associated with inherited dentine defects.

Location cDNA^a^	Protein	Ethnicity	Diagnosis	Mutation class	References
**Exon 2**					
C.16T > G g.16T > G	p.Y6D	Caucasian	DD-II	Missense	Rajpar et al., 2002
c.44C > T g.44C > T	p.A15V	Caucasian	DGI-II	Missense	Malmgren et al., 2004
c.49C > A g.49C > A	p.P17T	Chinese	DGI-II	Missense	Xiao et al., 2001
c.49C > T g.49C > T	p.P17S	Chinese	DGI-II	Missense	Zhang et al., 2007
		Caucasian	DGI-II	Missense	Hart et al., 2007
		Caucasian	DGI-II	Missense	McKnight *et al.*, 2008
c.50C > T g.50 > T	p.P17L	Chinese	DGI-II	Missense	Li et al., 2012
		Korean	DGI-II	Missense	Lee et al., 2013
**Intron 2**					
C.52-6T > G g.1185T > G IVS2-6T > G	p.V18_Q45del	Korean	DGI-II	Splice site	Lee et al., 2008
C.52-3C > G	p.V18_Q45del	Korean	DGI-II	Splice site	Kim et al., 2004
g.1188C > G IVS2-3C > G		Korean	DGI-II	Splice site	Kim et al., 2004
		Caucasian	DGI-II	Splice site	Kim et al., 2004
C.52-3C > A g.1194C > A IVS2-3		Finnish	DGI-II	Splice site	Holappa et al., 2006
c.52-25del23bp IVS2-3C-A		Chinese	DGI-II	Splice site	Wang et al., 2009
C.52-1G > A		Chinese	DGI-II	Splice site	Liu et al., 2016
C.52-2A > G	p.V18_Q45del	Chinese	DGI-III	Splice site	Li et al., 2017
		Thai	DGI-II	Missense	Porntaveetus et al., 2019
**Exon 3**					
c.52G > T g.1191G > T	p.V18F	Chinese	DGI-II	Missense	Xiao et al., 2001
		Korean	DGI-III	Missense	Kim et al., 2005
		Caucasian	DGI-III	Missense	Kim et al., 2005
		Chinese	DGI-II	Missense	Song *et al.*, 2006
		Finnish	DGI-II	Missense	Holappa et al., 2006
		Chinese	DGI-II	Missense	Li et al., 2017
c.53T > A g.1192T > A	p.V18D	Japanese	DGI-II	Missense	Kida et al., 2009
		Korean	DGI-II	Missense	Kim *et al.*, 2009
		Korean	DGI-II	Missense	Kim *et al.*, 2011
C.133C > T g.1272C > T	p.Q45X	Chinese	DGI-II	Missense	Zhang et al., 2001
		Chinese	DGI-II	Missense	Song *et al.*, 2006
**Intron 3**					
c.135 + 2T > C g.8662 T > C		Chinese	DGI-II	Splice site	Zhang et al., 2011
c.135 + 1G g.1275G > A IVS3 + 1G > A	p.V18_Q45del	Chinese	DGI-II	Splice site	Xiao et al., 2001
c.135 + 1G > T	p.V18_Q45del	Caucasian	DGI-II	Splice site	McKnight *et al*, 2008
c.135 + 3A > G IVS3 + 3A > G		Mongolian	DGI-II	Splice site	Bai et al., 2010
**Exon 4**					
c.202A > T g.1474A > T	p.R68W	Caucasian	DGI-II	Missense	Malmgren et al., 2004
		Finnish	DGI-II	Missense	Holappa et al., 2006

a Numbering assumes the A of the ATG start codon as nucleotide 1. Reference sequence NM_014208.3. c. = cDNA; g. = genomic; p. = protein; del = deletion; IVS = intervening sequence.

**Table 2.** Summary of *DPP* mutations associated with inherited dentin defects.

Location cDNA^a^	Protein	Ethnicity	Diagnosis	Mutation class	Reference
**Exon 5**					
c.1686delT	p.D562EfsX752	Finnish	DD-II	Frameshift	Nieminen et al., 2011
c.1830delC	p.S610RfsX704	French	DD-II	Frameshift	Nieminen et al., 2011
			DD-II	Frameshift	McKnight *et al.*, 2008
c.1870_1873delTCAG	p.S624TfsX687	Caucasian	DD-II	Frameshift	McKnight *et al.*, 2008^b^
c.1874_1877delACAG	p.D625AfsX687	Chinese	DDI-II	Frameshift	Li et al., 2017
c.1915_1918delAAGT	p.K639QfsX67	Thai	DGI	Frameshift	Porntaveetus *et al*, 2017
c.1918_1921delTCAG	p.S640TfsX671	Caucasian	DD-II	Frameshift	McKnight *et al.*, 2008^b^
c.1918_1921delTCAG	p.S640TfsX673	Greek	DD-II	Frameshift	Nieminen et al., 2011
c.1922_1925delACAG	p.D641AfsX672	Finnish	DD-II	Frameshift	Nieminen et al., 2011
c.2040delC	p.S680fsX1313	Chinese	DD-II	Frameshift	Song et al., 2008
c.2063delA	p.D688VfsX626	Finnish	DD-II	Frameshift	Nieminen et al., 2011
c.2134delA	p.S712AfsX602	Turkish	DD-II	Frameshift	Lee et al., 2019
c.2272delA	p.S758AfsX554	Caucasian	DGI-II/III	Frameshift	McKnight *et al.*, 2008^b^
c.2349delT	p.S783RfsX531	Spanish	DDI-II	Frameshift	Nieminen et al., 2011
c.2525delG	p.S842TfsX471	Caucasian	DGI-II/III	Frameshift	McKnight *et al.*, 2008^b^
c.2593delA	p.S865fsx1313	Chinese	DDI-II	Frameshift	Song et al., 2008
c.2666delG	p.S889TfsX425	Greek	DDI-II	Frameshift	Nieminen et al., 2011
c.2684delG	p.S895fsx1313	Chinese	DDI-II	Frameshift	Song et al., 2008
		Chinese	DDI-II	Frameshift	Li et al., 2017
c.2688delT	p.N896EfsX418	Korean	DDI-II	Frameshift	Lee *et al.*, 2010
c.3085A>G	p.N1029D	Chinese	NSHFb	Missense	Li et al., 2018
c.3087C>T	p.N1029D	Chinese	NSHFb	Missense	Li et al., 2018
c.3135delC	p.S1045RfsX269	Caucasian	DD-II	Frameshift	McKnight *et al.*, 2008^a^
c.3141delC		Caucasian	DD-II	Frameshift	McKnight *et al.*, 2008^b^
c.3179delG	p.S1060TfsX254	Korean	DD-II	Frameshift	Lee et al., 2019
c.3438delC	p.D1146fsX1313	Chinese	DDI-II	Frameshift	Song et al., 2008
c.3480_3481insCTGCT	p.D1161LfsX155	Korean	DD-II	Frameshift	Lee et al., 2019
c.3504_3508dup	p.D1170AfsX146	Chinese	DDI-II	Frameshift	Yang et al., 2015
c.3509_3521del13bp	p.D1170AfsX139	Chinese	DDI-II	Frameshift	Li et al., 2017
c.3546_3550delTAGCAinsG	p.D1182EfsX1312	Chinese	DDI-II	Frameshift	Song et al., 2008
c.3560delG	p.S1187MfsX127	Korean	DDI-II	Frameshift	Lee et al., 2011
c.3582_3591del10bp	p.D1194EfsX117 delCAGCAGCGAT	Finnish	DDI-II	Frameshift	Nieminen et al., 2011
c.3599_3634del36bp c.3715_3716ins18bp del1160_1171 ins1198_1199		American	DGI-III	Frameshift	Dong et al., 2005
c.3625_3700del76bp	p.D1209AfsX80	Vietnamese	DDI-II	Frameshift	Nieminen et al., 2011

a Numbering assumes the A of the ATG start codon as nucleotide 1. Reference sequence eNM_014208.3.

b NSHL, familial nonsyndromic hearing loss. c. = cDNA; p. = protein; fs = frameshift; del = deletion; ins = insert.

## List of Abbreviations

ALP: alkaline phosphatase
Amg: amelogenin
ASC: adult stem cell
BM: bone marrow
BMP: bone morphogenetic protein
BSP: bone sialoprotein
CAMKII: calmodulin-dependent protein kinase II
CCD: cleidocranial dysplasia
Col1α1: collagen type I alpha 1
DD: dentine dysplasia
DD-I: DD type 1
DD-II: DD type 2
DGI: dentineogenesis imperfecta
DGI-II: DGI type 2
DGI-III: DGI type 3
DGP: dentine glycoprotein
DMP1: dentine matrix protein 1
DPC: dental pulp cell
DPP: dentine phosphoprotein
DPSC: dental pulp stem cell
DSP: dentine sialoprotein
DSPP: dentine sialophosphoprotein
ECM: extracellular matrix
ER: endoplasmic reticulum
Erk1/2: extracellular signal-regulated kinase1/2
Erv29p: ER-derived vesicles protein 29
ESC: embryonic stem cell
FAK: focal adhesion kinase
GFAP: glial fibrillary acidic protein
HA: hydroxyapatite
hBMSC: human bone marrow stem cell
HSC: haematopoietic stem cell
iPSC: induced pluripotent stem cell
KO: knockout
MAPK: mitogen-activated protein kinase
MEPE: matrix extracellular phosphoglycoprotein
MMP: matrix metalloproteinase
MSC: mesenchymal stem cell
NCP: non-collagenous protein
Ocln: occludin
Ocn: osteocalcin
OPN: osteopontin
Osx: osterix
PP: phosphoryn
RD: reactionary dentine
rER: rough ER
RGD: arginine-glycine-aspartic acid
Runx2: runt-related transcription factor 2
SAPK/JNK: stress-activated protein kinase/Junamino-terminal kinase
SBE: Smad binding element
SCAPs: stem cells from the apical part of the papilla
SDS-PAGE: sodium dodecyl sulphate-polyaaylamide gel electrophoresis
SHED: human exfoliated deciduous teeth
SIBLING: small integrin-binding ligand N-linked glycoprotein
Surf4: surfeit locus protein 4
Tg: transgenic
TGF-β: transforming growth factor beta
TLR: Tolloid-like
UTR: untranslated region
WHO: World Health Organization

## References

[R1] Agnihotri R, Crawford HC, Haro H, Matrisian LM, Havrda MC, Liaw L (2001) Osteopontin, a novel substrate for matrix metalloproteinase-3 (stromelysin-1) and matrix metalloproteinase-7 (matrilysin). J Biol Chem 276: 28261–28267. 11375993 10.1074/jbc.M103608200

[R2] Alvares K, Stern PH, Arthur Veis A (2013) Dentin phosphoprotein binds annexin 2 and is involved in calcium transport in rat kidney ureteric bud cells. J Biol Chem 288:13036–13045. 23525114 10.1074/jbc.M112.389627 PMC3642346

[R3] Askar H, Krois J, Göstemeyer G, Schwendicke F (2021) Secondary caries risk of different adhesive strategies and restorative materials in permanent teeth: systematic review and network meta-analysis. J Dent 104:103541. DOI: 10.1016/j.jdent.2020.103541. 33259888

[R4] Bai H, Agula H, Wu Q, Zhou W, Sun Y, Qi Y, Latu S, Chen Y, Mutu J, Qiu C (2010) A novel DSPP mutation causes dentinogenesis imperfecta type II in a large mongolian family. BMC Med Genet 11:23. DOI: 10.1186/1471-2350-11-23. 20146806 PMC2829541

[R5] Baker JL, Morton JT, Dinis M, Alvarez R, Tran NC, Knight R, Edlund A (2021) Deep metagenomics examines the oral microbiome during dental caries, revealing novel taxa and co-occurrences with host molecules. Genome Res 31: 64–74. 33239396 10.1101/gr.265645.120 PMC7849383

[R6] Bellahcène A, Castronovo V, Ogbureke KUE, Fisher LW, Fedarko NS (2008) Small integrin-binding ligand N-linked glycoproteins (SIBLINGS): multifunctional proteins in cancer. Nat Rev Cancer 8: 212–226. 18292776 10.1038/nrc2345 PMC2484121

[R7] Bento L, Zhang Z, Imai A, Nör F, Dong Z, Shi S, Araujo F, Nör J (2013) Endothelial differentiation of SHED requires MEK1/ERK signaling. J Dent Res 92: 51–57. 23114032 10.1177/0022034512466263 PMC3521451

[R8] Bernabe E, Marcenes W, Hernandez CR, Bailey J, Abreu LG, Alipour V, Amini S, Arabloo J, Arefi Z, Arora A, Ayanore MA, Bärnighausen TW, Bijani A, Cho DY, Chu DT, Crowe CS, Demoz GT, Demsie DG, Dibaji Forooshani ZS, Du M, El Tantawi M, Fischer F, Folayan MO, Futran ND, Geramo YCD, Haj-Mirzaian A, Hariyani N, Hasanzadeh A, Hassanipour S, Hay SI, Hole MK, Hostiuc S, Ilic MD, James SL, Kalhor R, Kemmer L, Keramati M, Khader YS, Kisa S, Kisa A, Koyanagi A, Lalloo R, Le Nguyen Q, London SD, Manohar ND, Massenburg BB, Mathur MR, Meles HG, Mestrovic T, Mohammadian-Hafshejani A, Mohammadpourhodki R, Mokdad AH, Morrison SD, Nazari J, Nguyen TH, Nguyen CT, Nixon MR, Olagunju TO, Pakshir K, Pathak M, Rabiee N, Rafiei A, Ramezanzadeh K, Rios-Blancas MJ, Roro EM, Sabour S, Samy AM, Sawhney M, Schwendicke F, Shhmadi F, Shaikh MA, Stein C, Tovani-Palone MR, Tran BX, Unnikrishnan B, Vu GT, Vukovic A, Warouw TSS, Zaidi Z, Zhang ZJ, Kassebaum NJ (2020) Global, regional, and national levels and trends in burden of oral conditions from 1990 to 2017: a systematic analysis for the global burden of disease 2017 study. J Dent Res 99: 362–373. 32122215 10.1177/0022034520908533 PMC7088322

[R9] Bluteau G, Luder HU, De Bari C, Mitsiadis TA (2008) Stem cells for tooth engineering. Eur Cell Mater 16:1–9. 18671204 10.22203/ecm.v016a01

[R10] Burke FJ, Lucarotti PS (2009) How long do direct restorations placed within the general dental services in England and Wales survive? Br Dent J 206:1–5. 19057561 10.1038/sj.bdj.2008.1042

[R11] Cable J, Fuchs E, Weissman I, Jasper H, Glass D, Rando TA, Blau H, Debnath S, Oliva A, Park S, Passegué E, Kim C, Krasnow MA (2020) Adult stem cells and regenerative medicine-a symposium report. Ann N Y Acad Sci 1462: 27–36. 31655007 10.1111/nyas.14243 PMC7135961

[R12] Chai Y, Jiang X, Ito Y, Bringas P, Han J, Rowitch DH, Soriano P, McMahon AP, Sucov HM (2000) Fate of the mammalian cranial neural crest during tooth and mandibular morphogenesis. Development 127: 1671–1679. 10725243 10.1242/dev.127.8.1671

[R13] Chaplet M, Waltregny D, Detry C, Fisher LW, Castronovo V, Bellahcène A (2006) Expression of dentin sialophosphoprotein in human prostate cancer and its correlation with tumor aggressiveness. Int J Cancer 118: 850–856. 16108038 10.1002/ijc.21442

[R14] Chen H, Gu L, Liao B, Zhou X, Cheng L, Ren B (2020) Advances of anti-caries nanomaterials. Molecules 25:5047. DOI: 10.3390/molecules25215047. PMC7662703 33143140

[R15] Chen S, Rani S, Wu Y, Unterbrink A, Gu TT, Gluhak-Heinrich J, Chuang HH, Macdougall M (2005) Differential regulation of dentin sialophosphoprotein expression by Runx2 during odontoblast cytodifferentiation. J Biol Chem 280: 29717–29727. 15980071 10.1074/jbc.M502929200

[R16] Chen S, Gluhak-Heinrich J, Martinez M, Li T, Wu Y, Chuang HH, Chen L, Dong J, Gay I, MacDougall M (2008) Bone morphogenetic protein 2 mediates dentin sialophosphoprotein expression and odontoblast differentiation *via* NF-Y signaling. J Biol Chem 283: 19359–19370. 18424784 10.1074/jbc.M709492200 PMC2443643

[R17] Chen S, Gluhak-Heinrich J, Wang YH, Wu YM, Chuang HH, Chen L, Yuan GH, Dong J, Gay I, MacDougall M (2009). Runx2, Osx and Dspp in tooth development. J Dental Res 88: 904–909. 10.1177/0022034509342873 PMC3045537 19783797

[R18] Chen XD, Dusevich V, Feng JQ, Manolgas SC, Jilka RL (2007) Extracellular matrix made by bone marrow cells facilitates expansion of marrow-derived mesenchymal progenitor cells and prevents their differentiation into osteoblasts. J Bone Miner Res 22: 1943–1956. 17680726 10.1359/jbmr.070725

[R19] Chung HY, Li CC, Chia-Chan Hsu CC (2012) Characterization of the effects of 3DSS peptide on remineralized enamel in artificial saliva. J Mech Behav Biomed Mater 6: 74–79. 22301175 10.1016/j.jmbbm.2011.10.008

[R20] Cong X, Kong W (2020) Endothelial tight junctions and their regulatory signaling pathways in vascular homeostasis and disease. Cell Signal 66:109485. DOI: 10.1016/j.cellsig.2019.109485. 31770579

[R21] Couve E, Osorio R, Schmachtenberg O (2014) Reactionary dentinogenesis and neuroimmune response in dental caries. J Dent Res 93: 788–793. 24928097 10.1177/0022034514539507 PMC4293760

[R22] d’Aquino R, De Rosa A, Lanza V, Tirino V, Laino L, Graziano A, Desiderio V, Laino G, Papaccio G (2009) Human mandible bone defect repair by the grafting of dental pulp stem/progenitor cells and collagen sponge biocomplexes. Eur Cell Mater 18: 75–83. 19908196 10.22203/ecm.v018a07

[R23] de La Dure-Molla M, Philippe Fournier B, Berdal A (2015) Isolated dentinogenesis imperfecta and dentin dysplasia: revision of the classification. Eur J Hum Genet 23: 445–51. 25118030 10.1038/ejhg.2014.159 PMC4666581

[R24] Dong J, Gu TT, Jeffords L, MacDougall M (2005) Dentin phosphoprotein compound mutation in dentin sialophosphoprotein causes dentinogenesis imperfecta type III. Am J Med Genet A 132A: 305–309. 15690376 10.1002/ajmg.a.30460

[R25] D’Souza RN, Cavender A, Sunavala G, Alvarez J, Ohshima T, Kulkarni AB, MacDougall M (1997) Gene expression patterns of murine dentin matrix protein 1 (Dmp1) and dentin sialophosphoprotein (DSPP) suggest distinct developmental functions *in vivo*. J Bone Miner Res 12: 2040–2049. 9421236 10.1359/jbmr.1997.12.12.2040

[R26] D’Souza RN, Aberg T, Gaikwad J, Cavender A, Owen M, Karsenty G, Thesleff I (1999) Cbfa1 is required for epithelial-mesenchymal interactions regulating tooth development in mice. Development 126: 2911–2920. 10357935 10.1242/dev.126.13.2911

[R27] Du J, Lu Y, Song M, Yang L, Liu J, Chen X, Ma Y, Wang Y (2020) Effects of ERK/p38 MAPKs signaling pathways on MTA-mediated osteo/odontogenic differentiation of stem cells from apical papilla: a *vitro* study. BMC Oral Health 20: 50. DOI: 10.1186/s12903-020-1016-x. 32050954 PMC7017546

[R28] Ducy P, Zhang R, Geoffroy V, Ridall AL, Karsenty G (1997) Osf2/Cbfa1: a transcriptional activator of osteoblast differentiation. Cell 89: 747–754. 9182762 10.1016/s0092-8674(00)80257-3

[R29] Eapen A, Ramachandran A, George A (2012) Dentin phosphoprotein (DPP) activates integrin-mediated anchorage-dependent signals in undifferentiated mesenchymal cells. J Biol Chem 287: 5211–5224. 22134916 10.1074/jbc.M111.290080 PMC3285302

[R30] Eapen A, Kulkarni R, Ravindran S, Ramachandran A, Sundivakkam P, Tiruppathi C, George A (2013) Dentin phosphophoryn activates Smad protein signaling through Ca2+-calmodulin-dependent protein kinase II in undifferentiated mesenchymal cells. J Biol Chem 288: 8585–8595. 23362283 10.1074/jbc.M112.413997 PMC3605677

[R31] Figueiredo P, Sipponen MH, Lintinen K, Correia A, Kiriazis A, Yli-Kauhaluoma J, Österberg M, George A, Hirvonen J, Kostiainen MA, Santos HA (2019) Preparation and characterization of dentin phosphophoryn derived peptide-functionalized lignin nanoparticles for enhanced cellular uptake. Small 15: e1901427. DOI: 10.1002/smll.201901427. 31062448 PMC8042775

[R32] Fisher LW, Fedarko NS (2003) Six genes expressed in bones and teeth encode the current members of the SIBLING family of proteins. Connect Tissue Res 44 Suppl 1: 33–40. 12952171

[R33] Friedenstein AJ, Chailakhjan RK, Lalykina KS (1970) The development of fibroblast colonies in monolayer cultures of guinea-pig bone marrow and spleen cells. Cell Tissue Kinet 3: 393–403. 5523063 10.1111/j.1365-2184.1970.tb00347.x

[R34] Furger KA, Allan AL, Wilson SM, Hota C, Vantyghem SA, Postenka CO, Al-Katib W, Chambers AF, Alan B Tuck AB (2003) β3 integrin expression increases breast carcinoma cell responsiveness to the malignancy-enhancing effects of osteopontin. Mol Cancer Res 1: 810–819. 14517343

[R35] Gibson MP, Liu Q, Zhu Q, Lu Y, Jani P, Wang X, Liu Y, Paine ML, Snead ML, Feng JQ, Qin C (2013) Role of the NH2-terminal fragment of dentin sialophosphoprotein in dentinogenesis. Eur J Oral Sci 121: 76–85. 23489896 10.1111/eos.12020 PMC3602929

[R36] Gibson MP, 1, Jani P, Wang X, Lu Y, Qin C (2014) Overexpressing the NH2-terminal fragment of dentin sialophosphoprotein (DSPP) aggravates the periodontal defects in DSPP knockout mice. J Oral Biosci 56:143–148. 25386098 10.1016/j.job.2014.06.003 PMC4224573

[R37] Goldberg M, Smith AJ (2004) Cells and extracellular matrices of dentin and pulp: a biological basis for repair and tissue engineering. Crit Rev Oral Biol Med 15:13–27. 14761897 10.1177/154411130401500103

[R38] Götz M, Huttner WB (2005) The cell biology of neurogenesis. Nat Rev Mol Cell Biol 6: 777–788. 16314867 10.1038/nrm1739

[R39] Griffin SO, Jones K, Gray SK, Malvitz DM, Gooch BF (2008) Exploring four-handed delivery and retention of resin-based sealants. J Am Dent Assoc 139: 281–289. 18310732 10.14219/jada.archive.2008.0157

[R40] Gronthos S, Mankani M, Brahim J, Robey PG, Shi S (2000) Postnatal human dental pulp stem cells (DPSCs) *in vitro* and *in vivo*. Proc Natl Acad Sci U S A 97:13625–13630. 11087820 10.1073/pnas.240309797 PMC17626

[R41] Gronthos S, Brahim J, Li W, Fisher LW, Cherman N, Boyde A, DenBesten P, Robey PG, Shi S (2002) Stem cell properties of human dental pulp stem cells. J Dent Res 81: 531–535. 12147742 10.1177/154405910208100806

[R42] Gulseren G, Tansik G, Garifullin R, Tekinay AB, Guler MO (2019) Dentin phosphoprotein mimetic peptide nanofibers promote biomineralization. Macromol Biosci 19: e1800080. DOI: 10.1002/mabi.201800080 29745025

[R43] Guo W, He Y, Zhang X, Lu W, Wang C, Yu H, Liu Y, Li Y, Zhou Y, Zhou J, Zhang M, Deng Z, Jin Y (2009) The use of dentin matrix scaffold and dental follicle cells for dentin regeneration. Biomaterials 30: 6708–6723. 19767098 10.1016/j.biomaterials.2009.08.034

[R44] Han S, Peng X, Ding L, Lu J, Liu Z, Wang K, Zhang L (2021) TVH-19, a synthetic peptide, induces mineralization of dental pulp cells *in vitro* and formation of tertiary dentin *in vivo*. Biochem Biophys Res Commun 534: 837–842. 33168184 10.1016/j.bbrc.2020.10.095

[R45] Hart PS, Hart TC (2007) Disorders of human dentin. Cells Tissues Organs 186: 70–77. 17627120 10.1159/000102682 PMC4617234

[R46] He G, Ramachandran A, Dahl T, George S, Schultz D, Cookson D, Veis A, George A (2005) Phosphorylation of phosphophoryn is crucial for its function as a mediator of biomineralization. J Biol Chem 280: 33109–33114. 16046405 10.1074/jbc.M500159200

[R47] Higashikawa F, Eboshida A, Yokosaki Y (2007) Enhanced biological activity of polymeric osteopontin. FEBS Lett 581: 2697–2701. 17531983 10.1016/j.febslet.2007.05.018

[R48] Holappa H, Nieminen P, Tolva L, Lukinm PL, Alaluusua S (2006) Splicing site mutations in dentin sialophosphoprotein causing dentinogenesis imperfecta type II. Eur J Oral Sd 114: 381–384. 10.1111/j.1600-0722.2006.00391.x 17026502

[R49] Hsu CC, Chung HY, Yang JM, Shi W, Wu B (2011) Influence of 8DSS peptide on nano-mechanical behavior of human enamel. J Dent Res 90: 88–92. 20974901 10.1177/0022034510381904

[R50] Huang CC, Narayanan R, Warshawsky N, Ravindran S (2018) Dual ECM biomimetic scaffolds for dental pulp regenerative applications. Front Physiol 9: 495. DOI: 10.3389/fphys.2018.00495. 29887803 PMC5981804

[R51] Jadlowiec J, Koch H, Zhang X, Campbell PG, Seyedain M, Sfeir C (2004) Phosphophoryn regulates the gene expression and differentiation of NIH3T3, MC3T3-E1, and human mesenchymal stem cells *via* the integrin/MAPK signaling pathway. J Biol Chem 279: 53323–53330. 15371433 10.1074/jbc.M404934200

[R52] Jadlowiec JA, Zhang X, Li J, Campbell PG, Sfeir C (2006) Extracellular matrix-mediated signaling by dentin phosphophoryn involves activation of the Smad pathway independent of bone morphogenetic protein. J Biol Chem 281: 5341–5347. 16326713 10.1074/jbc.M506158200

[R53] Jonsson M, Fredriksson S, Jontell M, Linde A (1978) Isoelectric focusing of the phosphoprotein of rat incisor dentin in ampholine and acid pH gradients. Evidence for carrier ampholyte protein complexes. J Chromatogr 21: 234–42. 10.1016/s0021-9673(00)92338-0 29908

[R54] Kang J, Fan W, Deng Q, He H, Huang F (2019) Stem cells from the apical papilla: a promising source for stem cell-based therapy. BioMed Res Int 2019: 6104738. DOI: 10.1155/2019/6104738. 30834270 PMC6374798

[R55] Kaukua N, Shahidi MK, Konstantinidou C, Dyachuk V, Kaucka M, Furlan A, An Z, Wang L, Hultman I, Ahrlund-Richter L, Blom H, Brismar H, Lopes NA, Pachnis V, Suter U, Clevers H, Thesleff I, Sharpe P, Ernfors P, Fried K, Adameyko I (2014) Glial origin of mesenchymal stem cells in a tooth model system. Nature 513: 551–554. 25079316 10.1038/nature13536

[R56] Kazeminia M, Abdi A, Shohaimi S, Jalali R, Vaisi-Raygani A, Salari N, Mohammadi M (2020) Dental caries in primary and permanent teeth in children’s worldwide, 1995 to 2019: a systematic review and meta-analysis. Head Face Med 16: 22. DOI: 10.1186/s13005-020-00237-z. 33023617 PMC7541284

[R57] Kida M, Tsutsumi T, Shindoh M, Ikeda H, Tadashi Ariga T (2009) *De novo* mutation in the DSPP gene associated with dentinogenesis imperfecta type II in a Japanese family. Eur J Oral Sd 117: 691–694. 10.1111/j.1600-0722.2009.00683.x 20121932

[R58] Kim JW, Nam SH, Jang KT, Lee SH, Kim CC, Hahn SH, Hu JC, Simmer JP (2004) A novel splice acceptor mutation in the DSPP gene causing dentinogenesis imperfecta type II. Hum Genet 115: 248–254. 15241678 10.1007/s00439-004-1143-5

[R59] Kim JW, Hu JC, Lee JI, Moon SK, Kim YJ, Jang KT, Lee SH, Kim CC, Hahn SH, Simmer JP (2005) Mutational hot spot in the DSPP gene causing dentinogenesis imperfeda type II. Hum Genet 116: 186–191. 15592686 10.1007/s00439-004-1223-6

[R60] Kobuke S, Suzuki S, Hoshino H, Haruyama N, Nishimura F, Shiba H (2015) Relationship between length variations in Ser/Asp-rich repeats in phosphophoryn and *in vitro* precipitation of calcium phosphate. Arch Oral Biol 60:1263–1272. 26099661 10.1016/j.archoralbio.2015.05.013

[R61] Lee B, Thirunavukkarasu K, Zhou L, Pastore L, Baldini A, Hecht J, Geoffroy V, Ducy P, Karsenty G (1997) Missense mutations abolishing DNA binding of the osteoblast-specific transcription factor OSF2/CBFA1 in cleidocranial dysplasia. Nat Genet 16:307–310. 9207800 10.1038/ng0797-307

[R62] Lee JH, Seo SJ (2016) Biomedical application of dental tissue-derived induced pluripotent stem cells. Stem Cells Int 2016: 9762465. DOI: 10.1155/2016/9762465. 26989423 PMC4773578

[R63] Lee JW, Hong J, Seymen F, Kim YJ, Kang J, Koruyucu M, Tuloglu N, Bayrak S, Song JS, Shin TJ, Hyun HK, Kim YJ, Lee JC, Park JC, Hu J, Simmer J, Kim JW (2019) Novel frameshift mutations in DSPP cause dentin dysplasia type II. Oral Dis 25:2044–2046. 31454439 10.1111/odi.13182 PMC7098805

[R64] Lee KE, Kang HY, Lee SK, Yoo SH, Lee JC, Hwang YH, Nam KH, Kim JS, Park JC, Kim JW (2011) Novel dentin phosphoprotein frameshift mutations in dentinogenesis imperfecta type II. Clin Genet 79: 378–384. 20618350 10.1111/j.1399-0004.2010.01483.x

[R65] Lee SK, Hu JCC, Lee KE, Simmer JP, Kim JW (2008) A dentin sialophosphoprotein mutation that partially disrupts a splice acceptor site causes type II dentin dysplasia. J Endod 34:1470–1473. 19026876 10.1016/j.joen.2008.08.027 PMC2763612

[R66] Lee SK, Lee KE, Hwang YH, Kida M, Tsutsumi T, Ariga T, Park JC, Kim JW (2011) Identification of the DSPP mutation in a new kindred and phenotype-genotype correlation. Oral Dis 17: 314–319. 21029264 10.1111/j.1601-0825.2010.01760.x

[R67] Lee SK, Lee KE, Song SJ, Hyun HK, Lee SH, Kim JW (2013) A DSPP mutation causing dentinogenesis imperfecta and characterization of the mutational effect. Biomed Res Int 2013: 948181. DOI: 10.1155/2013/948181. 23509818 PMC3591212

[R68] Lee SY, Kim SY, Park SH, Kim JJ, Jang JH, Kim EC (2012) Effects of recombinant dentin sialoprotein in dental pulp cells. J Dent Res 91:407–412. 22269273 10.1177/0022034511436113

[R69] Li D, Du X, Zhang R, Shen B, Huang Y, Valenzuela RK, Wang B, Zhao H, Liu Z, Li J, Xu Z, Gao L, Ma J (2012) Mutation identification of the DSPP in a Chinese family with DGI-II and an up-to-date bioinformatic analysis. Genomics 99: 220–226. 22310900 10.1016/j.ygeno.2012.01.006

[R70] Li F, Liu Y, Liu H, Yang J, Zhang F, Feng H (2017) Phenotype and genotype analyses in seven families with dentinogenesis imperfecta or dentin dysplasia. Oral Dis 23: 360–366. 27973701 10.1111/odi.12621

[R71] Li W, Chen L, Chen Z, Wu L, Feng J, Wang F, Shoff L, Li X, Donly KJ, MacDougall M, Chen S (2017) Dentin sialoprotein facilitates dental mesenchymal cell differentiation and dentin formation. Sci Rep 7: 300. DOI: 10.1038/s41598-017-00339-w. 28331230 PMC5428264

[R72] Li WX, Peng H, Yang L, Hao QQ, Sun W, Ji F, Guo WW, Yang SM (2018) Familial nonsyndromic hearing loss with incomplete partition type II caused by novel DSPP gene mutations. Acta Otolaryngol 138:685–690. 29741433 10.1080/00016489.2018.1459832

[R73] Liang T, Zhang H, Xu Q, Wang S, Qin C, Lu Y (2019) Mutant dentin sialophosphoprotein causes dentinogenesis imperfecta. J Dent Res 98: 912–919. 31173534 10.1177/0022034519854029 PMC6616118

[R74] Linde A, Goldberg M (1993) Dentinogenesis (review) Crit Rev Oral Biol Med 4: 679–728. 8292714 10.1177/10454411930040050301

[R75] Liu Y, Huang Y, Gao J, Li S, Zhao X, Zhang X (2016) Identification of a novel mutation of DSPP gene in a Chinese family affected with dentinogenesis imperfecta shields type II. Zhonghua Yi Xue Yi Chuan Xue Za Zhi 33: 34–37. 26829730 10.3760/cma.j.issn.1003-9406.2016.01.009

[R76] Lopez-Cazaux S, Bluteau G, Magne D, Lieubeau B, Guicheux J, Alliot-Licht B (2006) Culture medium modulates the behavior of human dental pulp-derived cells: technical note. Eur Cell Mater 11:35–42. 16485235 10.22203/ecm.v011a05

[R77] MacDougall M, Simmons D, Luan X, Nydegger J, Feng J, Gu TT (1997) Dentin phosphoprotein and dentin sialoprotein are cleavage products expressed from a single transcript coded by a gene on human chromosome 4. Dentin phosphoprotein DNA sequence determination. J Biol Chem 272: 835–842. 8995371 10.1074/jbc.272.2.835

[R78] Malmgren B, Lindskog S, Elgadi A, Norgren S (2004) Clinical, histopathologic, and genetic investigation in two large families with dentinogenesis imperfecta type II. Hum Genet 114: 491–498. 14758537 10.1007/s00439-004-1084-z

[R79] Marschall VZ, Fisher LW (2008) Dentin matrix protein-1 isoforms promote differential cell attachment and migration J Biol Chem 283: 32730–32740. 18819913 10.1074/jbc.M804283200 PMC2583300

[R80] Marschall VZ, Fisher LW (2010) Dentin sialophosphoprotein (DSPP) is cleaved into its two natural dentin matrix products by three isoforms of bone morphogenetic protein-1 (BMP1). Matrix Biol 29: 295–303. 20079836 10.1016/j.matbio.2010.01.002 PMC2862847

[R81] Martens W, Sanen K, Georgiou M, Struys T, Bronckaers A, Ameloot M, Phillips J, Lambrichts I (2014) Human dental pulp stem cells can differentiate into Schwann cells and promote and guide neurite outgrowth in an aligned tissue-engineered collagen construct *in vitro*. FASEB J 28:1634–1643. 24352035 10.1096/fj.13-243980 PMC4046066

[R82] McKnight DA, Simmer JP, Hart PS, Hart TC, Fisher LW (2008a) Overlapping DSPP mutations cause dentin dysplasia and dentinogenesis imperfecta. J Dent Res 87:1108–1111. 19029076 10.1177/154405910808701217 PMC2596760

[R83] McKnight DA, Hart PS, Hart TC, Hartsfield JK, Wilson A, Wright JT, Fisher LW (2008b) A comprehensive analysis of normal variation and disease-causing mutations in the human DSPP gene. Hum Mutat 29:1392–404. 18521831 10.1002/humu.20783 PMC5534847

[R84] Méndez-Ferrer S, Bonnet D, Steensma DP, Hasserjian RP, Ghobrial IM, Gribben JG, Andreeff M, Krause DS (2020) Bone marrow niches in haematological malignancies. Nat Rev Cancer 20: 285–298. 32112045 10.1038/s41568-020-0245-2 PMC9912977

[R85] Miran S, Mitsiadis TA, Pagella P (2016) Innovative dental stem cell-based research approaches: the future of dentistry. Stem Cells Int 2016:7231038. DOI: 10.1155/2016/7231038. 27648076 PMC5018320

[R86] Mitsiadis TA, Orsini G, Jimenez-Rojo L (2015) Stem cell-based approaches in dentistry. Eur Cell Mater 30: 248–257. 26562631 10.22203/ecm.v030a17

[R87] Miura M, Gronthos S, Zhao M, Lu B, Fisher LW, Robey PG, Shi S (2003) SHED: stem cells from human exfoliated deciduous teeth. Proc Natl Acad Sci U S A 100: 5807–5812. 12716973 10.1073/pnas.0937635100 PMC156282

[R88] Miyazaki T, Kanatani N, Rokutanda S, Yoshida C, Toyosawa S, Nakamura R, Takada S, Komori T (2008) Inhibition of the terminal differentiation of odontoblasts and their trans-differentiation into osteoblasts in Runx2 transgenic mice. Arch Histol Cytol 71:131–146. 18974605 10.1679/aohc.71.131

[R89] Morrison SJ, Spradling AC (2008) Stem cells and niches: mechanisms that promote stem cell maintenance throughout life. Cell 132: 598–611. 18295578 10.1016/j.cell.2008.01.038 PMC4505728

[R90] Morsczeck C, Gotz W, Schierholz J, Zeilhofer F, Kuhn U, Mohl C, Sippel C, Hoffmann KH (2005) Isolation of precursor cells (PCs) from human dental follicle of wisdom teeth. Matrix Biol 24:155–165. 15890265 10.1016/j.matbio.2004.12.004

[R91] Nada OA, El Backly RM (2018) Stem cells from the apical papilla (SCAP) as a tool for endogenous tissue regeneration. Front Bioeng Biotechnol 6: 103. DOI: 10.3389/fbioe.2018.00103. 30087893 PMC6066565

[R92] Nakashima M (2005) Bone morphogenetic proteins in dentin regeneration for potential use in endodontic therapy. Cytokine Growth Factor Rev 16: 369–376. 15878301 10.1016/j.cytogfr.2005.02.011

[R93] Nanci A (2012) Ten Cate’s oral histology: development, structure, and function (8th edition). Mosby, USA. pp: 1–13.

[R94] Neves VCM, Sharpe PT (2018) Regulation of reactionary dentine formation. J Dent Res 97:416–422. 29185832 10.1177/0022034517743431

[R95] Ni SL, Zhang J, Liu X, Li XW, Sun YJ, Zhang X, Wang L, Lu JJ, Cui Y, Zheng CY, Han B, Sun HC (2018) Effects of human bone morphogenetic protein 2 (hBMP2) on tertiary dentin formation. Am J Transl Res 10: 2868–2876. 30323873 PMC6176225

[R96] Nieminen P, Papagiannoulis-Lascarides L, Waltimo-Siren J, Ollila P, Karjalainen S, Arte S, Veerkamp J, Walton VT, Küstner EC, Siltanen T, Holappa H, Lukinm PL, Alaluusua S (2011) Frameshift mutations in dentin phosphoprotein and dependence of dentin disease phenotype on mutation location. J Bone Miner Res 26: 873–880. 20949630 10.1002/jbmr.276

[R97] Ozer A, Yuan GH, Yang GB, Wang F, Li W, Yang Y, Guo F, Gao QP, Shoff L, Chen Z, Gay I, Donly KJ, MacDougall M, Chen S (2013) Domain of dentine sialoprotein mediates proliferation and differentiation of human periodontal ligament stem cells. Plos One 8: e81655. DOI: 10.1371/journal.pone.0081655. 24400037 PMC3882282

[R98] Paine ML, Luo W, Wang HJ, Bringas P Jr, Ngan AY, Miklus VG, Zhu DH, MacDougall M, White SN, Snead ML (2005) Dentin phosphoprotein overexpression during amelogenesis. J Biol Chem 280: 31991–31998. 16014627 10.1074/jbc.M502991200

[R99] Perry JM, Li L (2007) Disrupting the stem cell niche: good seeds in bad soil. Cell 129:1045–1047. 17574018 10.1016/j.cell.2007.05.053

[R100] Pisciotta A, Riccio M, Carnevale G, Lu A, De Biasi S, Gibellini L, La Sala GB, Bruzzesi G, Ferrari A, Huard J, De Pol A (2015) Stem cells isolated from human dental pulp and amniotic fluid improve skeletal muscle histopathology in mdx/SCID mice. Stem Cell Res Ther 6:156. DOI: 10.1186/s13287-015-0141-y. 26316011 PMC4552417

[R101] Pittenger MF, Mackay AM, Beck SC, Jaiswal RK, Douglas R, Mosca JD, Moorman MA, Simonetti DW, Craig S, Marshak DR (1999) Multilineage potential of adult human mesenchymal stem cells. Science 284: 143–147. 10102814 10.1126/science.284.5411.143

[R102] Porntaveetus T, Nowwarote N, Osathanon T, Theerapanon T, Pavasant P, Boonprakong L, Sanon K, Srisawasdi S, Suphapeetiporn K, Shotelersuk V (2019) Compromised alveolar bone cells in a patient with dentinogenesis imperfecta caused by DSPP mutation. Clin Oral Investig 23: 303–313. 10.1007/s00784-018-2437-7 29679229

[R103] Prince CW, Dickie D, Krumdieck CL (1991) Osteopontin, a substrate for transglutaminase and factor XIII activity. Biochem Biophys Res Commun 177:1205–1210. 1676261 10.1016/0006-291x(91)90669-x

[R104] Qin C, Cook RG, Orkiszewski RS, Butler WT (2001) Identification and characterization of the carboxyl-terminal region of rat dentin sialoprotein. J Biol Chem 276: 904–909. 11042175 10.1074/jbc.M006271200

[R105] Qin C, Brunn JC, Cadena E, Ridall A, Tsujigiwa H, Nagatsuka H, Nagai N, Butler WT (2002) The expression of dentin sialophosphoprotein gene in bone. J Dent Res 81: 392–394. 12097430 10.1177/154405910208100607

[R106] Rajpar MH, Koch MJ, Davies RM, Mellody KT, Kielty CM, Dixon MJ (2002) Mutation of the signal peptide region of the bicistronic gene DSPP affects translocation to the endoplasmic reticulum and results in defective dentine biomineralization. Hum Mol Genet 11: 2559–2565. 12354781 10.1093/hmg/11.21.2559

[R107] Rangaswami H, Bulbule A, Kundu GC (2006) Osteopontin: role in cell signaling and cancer progression. Trends Cell Biol 16: 79–87. 16406521 10.1016/j.tcb.2005.12.005

[R108] Ravindran S, Snee PT, Ramachandran A, George A (2013) Acidic domain in dentin phosphophoryn facilitates cellular uptake. J Biol Chem 288:16098–109. 23589294 10.1074/jbc.M113.450585 PMC3668765

[R109] Ruch JV (1985) Odontoblast differentiation and the formation of the odontoblast layer. J Dent Res 64: 489–498. 3857251 10.1177/002203458506400402

[R110] Saoud TMA, Ricucci D, Lin LM, Gaengler P (2016) Regeneration and repair in endodontics-a special issue of the regenerative endodontics-a new era in clinical endodontics. Dent J (Basel) 4:3. DOI: 10.3390/dj4010003. PMC5851202 29563445

[R111] Seo BM, Miura M, Gronthos S, Bartold PM, Batouli S, Brahim J, Young M, Robey PG, Wang CY, Shi S (2004) Investigation of multipotent postnatal stem cells from human periodontal ligament. Lancet 364: 149–155. 15246727 10.1016/S0140-6736(04)16627-0

[R112] Shi S, Gronthos S (2003) Perivascular niche of postnatal mesenchymal stem cells in human bone marrow and dental pulp. J Bone Miner Res 18: 696–704. 12674330 10.1359/jbmr.2003.18.4.696

[R113] Sonoyama W, Liu Y, Yamaza T, Tuan RS, Wang S, Shi S, Huang GT (2008) Characterization of the apical papilla and its residing stem cells from human immature permanent teeth: a pilot study. J Endod 34: 166–171. 18215674 10.1016/j.joen.2007.11.021 PMC2714367

[R114] Song YL, Wang CN, Fan MW, Su B, Z Bian Z (2008) Dentin phosphoprotein frameshift mutations in hereditary dentin disorders and their variation patterns in normal human population. J Med Genet 45: 457–464. 18456718 10.1136/jmg.2007.056911

[R115] Sreenath T, Thyagarajan T, Hall B, Longenecker G, D’Souza R, Hong S, Wright JT, MacDougall M, Sauk J, Ashok B Kulkarni AB (2003) Dentin sialophosphoprotein knockout mouse teeth display widened predentin zone and develop defective dentin mineralization similar to human dentinogenesis imperfecta type III. J Biol Chem 278: 24874–24880. 12721295 10.1074/jbc.M303908200

[R116] Steiglitz BM, Ayala M, Narayanan K, George A, Greenspan DS (2004) Bone morphogenetic protein-1/Tolloid-like proteinases process dentin matrix protein-1. J Biol Chem 279: 980–986. 14578349 10.1074/jbc.M310179200

[R117] Sui B, Chen C, Kou X, Li B, Xuan K, Shi S, Jin Y (2019) Pulp stem cell-mediated functional pulp regeneration. J Dent Res 98: 27–35. 30372659 10.1177/0022034518808754

[R118] Suzuki S, Sreenath T, Haruyama N, Honeycutt C, Terse A, Cho A, Kohler T, Müller R, Goldberg M, Kulkarni AB (2009) Dentin sialoprotein and dentin phosphoprotein have distinct roles in dentin mineralization. Matrix Biol 28: 221–229. 19348940 10.1016/j.matbio.2009.03.006 PMC2758621

[R119] Suzuki S, Kobuke S, Haruyama N, Hoshino H, Kulkarni AB, Nishimura F (2014) Adhesive and migratory effects of phosphophoryn are modulated by flanking peptides of the integrin binding motif. PLoS One 9: e112490. DOI: 10.1371/journal.pone.0112490. 25396425 PMC4232355

[R120] Suzuki S, Nakanishi J, Yoshida K, Shiba H (2016) Dentin sialophosphoprotein is a potentially latent bioactive protein in dentin. J Oral Biosci 58:134–142. 32512682 10.1016/j.job.2016.08.002

[R121] Teramoto H, Castellone MD, Malek RL, Letwin N, Frank B, Gutkind JS, Lee NH (2005) Autocrine activation of an osteopontin-CD44–Rac pathway enhances invasion and transformation by H-RasV12. Oncogene 24: 489–501. 15516973 10.1038/sj.onc.1208209

[R122] Thesleff I (2003) Epithelial-mesenchymal signaling regulating tooth morphogenesis. J Cell Sd 116:1647–1648. 10.1242/jcs.00410 12665545

[R123] Tirino V, Paino F, De Rosa A, Papaccio G (2012) Identification, isolation, characterization, and banking of human dental pulp stem cells. Methods Mol Biol 879: 443–463. 22610575 10.1007/978-1-61779-815-3_26

[R124] Tsuchiya S, Simmer JP, Hu JC, Richardson AS, Yamakoshi F, Yamakoshi Y (2011) Astacin proteases cleave dentin sialophosphoprotein (Dspp) to generate dentin phosphoprotein (Dpp). J Bone Miner Res 26: 220–228. 20687161 10.1002/jbmr.202 PMC3179315

[R125] Tziafas D, Smith AJ, Lesot H (2000) Designing new treatment strategies in vital pulp therapy. J Dent 28: 77–92. 10666965 10.1016/s0300-5712(99)00047-0

[R126] Vijaykumar A, Dyrkacz P, Vidovic-Zdrilic I, Maye P, Mina M (2020) Expression of BSP-GFPtpz transgene during osteogenesis and reparative dentinogenesis. J Dent Res 99: 89–97. 31682548 10.1177/0022034519885089 PMC6927219

[R127] Vidovic I, Banerjee A, Fatahi R, Matthews BG, Dyment NA, Kalajzic I, Mina M (2017) αSMA-expressing perivascular cells represent dental pulp progenitors *in vivo*. J Dent Res 96: 323–330. 27834664 10.1177/0022034516678208 PMC5298392

[R128] Waddington RJ, Youde SJ, Lee CP, Sloan AJ (2009) Isolation of distinct progenitor stem cell populations from dental pulp. Cells Tissues Organs 189:268–274. 18701814 10.1159/000151447

[R129] Walker JV, Zhuang H, Singer D, Illsley CS, Kok WL, Sivaraj KK, Gao Y, Bolton C, Liu Y, Zhao M, Grayson PRC, Wang S, Karbanová J, Lee T, Ardu S, Lai Q, Liu J, Kassem M, Chen S, Yang K, Bai Y, Tredwin C, Zambon AC, Corbeil D, Adams R, Abdallah BM, Hu B (2019) Transit amplifying cells coordinate mouse incisor mesenchymal stem cell activation. Nat Commun 10: 3596. DOI: 10.1038/S41467-019-11611-0. 31399601 PMC6689115

[R130] Wan C, Yuan G, Luo D, Zhang L, Lin H, Liu H, Chen L, Yang G, Chen S, Chen Z (2016) The dentin sialoprotein (DSP) domain regulates dental mesenchymal cell differentiation through a novel surface receptor. Sci Rep 6: 29666. DOI: 10.1038/srep29666. 27430624 PMC4949421

[R131] Wang HY, Hou YN, Cui YX, Huang YF, Shi YC, Xia XY, Lu HY, Wang YH, Li XJ (2009) A novel splice site mutation in the dentin sialophosphoprotein gene in a Chinese family with dentinogenesis imperfecta type II. Mutat Res 662: 22–27. DOI: 10.1016/j.mrfmmm.2008.11.019. 19103209

[R132] Wang L, Dorn P, Zeinali S, Froment L, Berezowska S, Kocher GJ, Alves MP, Brügger M, Esteves BIO, Blank F, Wotzkow C, Steiner S, Amacker M, Peng RW, Marti TM, Guenat OT, Bode PK, Moehrlen U, Schmid RA, Hall SRR (2020a) CD90(+)CD146(+) identifies a pulmonary mesenchymal cell subtype with both immune modulatory and perivascularlike function in postnatal human lung. Am J Physiol Lung Cell Mol Physiol 318: L813–L830. DOI: 10.1152/ajplung.00146.2019. 32073879

[R133] Wang Y, Zhu M, Zhu XX (2020b) Functional fillers for dental resin composites. Acta Biomater 122:50–65. 33290913 10.1016/j.actbio.2020.12.001

[R134] White SN, Paine ML, Ngan AYW, Miklus VG, Luo W, Wang HJ, Malcolm L. Snead ML (2007) Ectopic expression of dentin sialoprotein during amelogenesis hardens bulk enamel. J Biol Chem 282: 5340–5345. 17189271 10.1074/jbc.M604814200

[R135] Wilkinson AC, Igarashi KJ, Nakauchi H (2020) Haematopoietic stem cell self-renewal *in vivo* and *ex vivo*. Nat Rev Genet 21: 541–554. 32467607 10.1038/s41576-020-0241-0 PMC7894993

[R136] Witkop CJ Jr (1975) Hereditary defects of dentin. Dent Clin North Am 19: 25–45. 162890

[R137] Xiao S, Yu C, Chou X, Yuan W, Wang Y, Bu L, Fu G, Qian M, Yang J, Shi Y, Hu L, Han B, Wang Z, Huang W, Liu J, Chen Z, Zhao G, Kong X (2001) Dentinogenesis imperfecta 1 with or without progressive hearing loss is associated with distinct mutations in DSPP. Nat Genet 27: 201–204. 11175790 10.1038/84848

[R138] Yamakoshi Y, Hu JC, Fukae M, Zhang H, Simmer JP (2005) Dentin glycoprotein: the protein in the middle of the dentin sialophosphoprotein chimera. J Biol Chem 280:17472–17479. 15728577 10.1074/jbc.M413220200

[R139] Yamakoshi Y, Hu JC, Iwata T, Kobayashi K, Fukae M, Simmer JP (2006) Dentin sialophosphoprotein is processed by MMP-2 and MMP-20 *in vitro* and *in vivo*. J Biol Chem 281: 38235–38243. 17046814 10.1074/jbc.M607767200

[R140] Yamanaka S (2020) Pluripotent stem cell-based cell therapy-promise and challenges. Cell Stem Cell 27: 523–531. 33007237 10.1016/j.stem.2020.09.014

[R141] Yamaza T, Kentaro A, Chen C, Liu Y, Shi Y, Gronthos S, Wang S, Shi S (2010) Immunomodulatory properties of stem cells from human exfoliated deciduous teeth. Stem Cell Res Ther 1: 5. DOI: 10.1186/scrt5. 20504286 PMC2873699

[R142] Yang J, Kawasaki K, Lee M, Reid BM, Nunez SM, Choi M, Seymen F, Koruyucu M, Kasimoglu Y, Estrella-Yuson N, Lin BP, Simmer JP, Hu JC (2015) The dentin phosphoprotein repeat region and inherited defects of dentin. Mol Genet Genomic Med 4: 28–38. 26788535 10.1002/mgg3.176 PMC4707025

[R143] Yang JW, Shin YY, Seo Y, Kim HS (2020b) Therapeutic functions of stem cells from oral cavity: an update. Int J Mol Sci 21: 4389. DOI: 10.3390/ijms21124389. PMC7352407 32575639

[R144] Yang SY, Choi JW, Kim KM, Kwon JS (2020a) Prevention of secondary caries using resin-based pit and fissure sealants containing hydrated calcium silicate. Polymers (Basel) 12: 1200. DOI: 10.3390/polym12051200. PMC7284760 32466181

[R145] Yang X, Ma Y, Guo W, Yang B, Tian W (2019) Stem cells from human exfoliated deciduous teeth as an alternative cell source in bio-root regeneration. Theranostics 9: 2694–2711. 31131062 10.7150/thno.31801 PMC6525984

[R146] Yin Y, Garcia MR, Novak AJ, Saunders AM, Ank RS, Nam AS, Fisher LW (2018) Surf4 (Erv29p) binds amino-terminal tripeptide motifs of soluble cargo proteins with different affinities, enabling prioritization of their exit from the endoplasmic reticulum. PLoS Biol 16: e2005140. DOI: 10.1371/journal.pbio.2005140. 30086131 PMC6097701

[R147] Yuan G, Yang G, Song G, Chen Z, Chen S (2012) Immunohistochemical localization of the NH(2)-terminal and COOH-terminal fragments of dentin sialoprotein in mouse teeth. Cell Tissue Res 349: 605–614. 22581382 10.1007/s00441-012-1418-4 PMC3569491

[R148] Yuan G, Chen L, Feng JS, Yang G, Ni Q, Xu XP, Wan C, Lindsey M, Donly KJ, MacDougall M, Chen Z, Chen S (2017) Dentin sialoprotein is a novel substrate of matrix metalloproteinase 9 *in vitro* and *in vivo*. Sci Rep 7: 42449. DOI: 10.1038/srep42449. 28195206 PMC5307955

[R149] Zaugg LK, Banu A, Walther AR, Chandrasekaran D, Babb RC, Salzlechner C, Hedegrd MAB, Gentleman E, Sharpe PT (2020) Translation approach for dentine regeneration using GSK-3 antagonists. J Dent Res 99: 544–551. 32156176 10.1177/0022034520908593 PMC7534023

[R150] Zhang H, Liu P, Wang S, Liu C, Jani P, Lu Y, Qin C (2016) Transgenic expression of dentin phosphoprotein inhibits skeletal development. Eur J Histochem 60: 2587. DOI: 10.4081/ejh.2016.2587. 26972716 PMC4800252

[R151] Zhang H, Xie X, Liu P, Liang T, Lu Y, Chunlin Qin C (2018) Transgenic expression of dentin phosphoprotein (DPP) partially rescued the dentin defects of DSPP-null mice. PLoS One 13: e0195854. DOI: 10.1371/journal.pone.0195854. 29672573 PMC5908185

[R152] Zhang J, Wang J, Ma Y, Du W, Zhao S, Zhang Z, Zhang X, Liu Y, Xiao H, Wang H, Jin L, Liu J (2011) A novel splicing mutation alters DSPP transcription and leads to dentinogenesis imperfecta type II. PLoS One 6: e27982. DOI: 10.1371/journal.pone.0027982. 22125647 PMC3220712

[R153] Zhang X, Zhao J, Li C, Gao S, Qiu C, Liu P, Wu G, Qiang B, Lo WH, Shen Y (2001) DSPP mutation in dentinogenesis imperfecta shields type II. Nat Genet 27:151–152. 11175779 10.1038/84765

[R154] Zhang X, Chen L, Liu J, Zhao Z, Qu E, Wang X, Chang W, Xu C, Wang QK, Liu M (2007) A novel DSPP mutation is associated with type II dentinogenesis imperfecta in a Chinese family. BMC Med Genet 8: 52. DOI: 10.1186/1471-2350-8-52. 17686168 PMC1995191

[R155] Zhu Q, Gibson MP, Liu Q, Liu Y, Lu Y, Wang X, Feng JQ, Qin C (2012) Proteolytic processing of dentin sialophosphoprotein (DSPP) is essential to dentinogenesis. J Biol Chem 287: 30426–30435. 22798071 10.1074/jbc.M112.388587 PMC3436292

